# Bridging the sim2real gap. Investigating deviations between experimental motion measurements and musculoskeletal simulation results—a systematic review

**DOI:** 10.3389/fbioe.2024.1386874

**Published:** 2024-06-11

**Authors:** Iris Wechsler, Alexander Wolf, Julian Shanbhag, Sigrid Leyendecker, Bjoern M. Eskofier, Anne D. Koelewijn, Sandro Wartzack, Jörg Miehling

**Affiliations:** ^1^ Engineering Design, Department of Mechanical Engineering, Friedrich-Alexander-Universität Erlangen-Nürnberg, Erlangen, Germany; ^2^ Institute of Applied Dynamics, Department of Mechanical Engineering, Friedrich-Alexander-Universität Erlangen-Nürnberg, Erlangen, Germany; ^3^ Machine Learning and Data Analytics Lab, Department Artificial Intelligence in Biomedical Engineering (AIBE), Friedrich-Alexander-Universität Erlangen-Nürnberg, Erlangen, Germany; ^4^ Chair of Autonomous Systems and Mechatronics, Department of Electrical Engineering, Friedrich-Alexander-Universität Erlangen-Nürnberg, Erlangen, Germany

**Keywords:** systematic review, biomechanical modeling and simulation, sim2real gap, data tracking methods, residual minimisation, inverse dynamics, forward dynamic simulations

## Abstract

Musculoskeletal simulations can be used to estimate biomechanical variables like muscle forces and joint torques from non-invasive experimental data using inverse and forward methods. Inverse kinematics followed by inverse dynamics (ID) uses body motion and external force measurements to compute joint movements and the corresponding joint loads, respectively. ID leads to residual forces and torques (residuals) that are not physically realistic, because of measurement noise and modeling assumptions. Forward dynamic simulations (FD) are found by tracking experimental data. They do not generate residuals but will move away from experimental data to achieve this. Therefore, there is a gap between reality (the experimental measurements) and simulations in both approaches, the sim2real gap. To answer (patho-) physiological research questions, simulation results have to be accurate and reliable; the sim2real gap needs to be handled. Therefore, we reviewed methods to handle the sim2real gap in such musculoskeletal simulations. The review identifies, classifies and analyses existing methods that bridge the sim2real gap, including their strengths and limitations. Using a systematic approach, we conducted an electronic search in the databases Scopus, PubMed and Web of Science. We selected and included 85 relevant papers that were sorted into eight different solution clusters based on three aspects: how the sim2real gap is handled, the mathematical method used, and the parameters/variables of the simulations which were adjusted. Each cluster has a distinctive way of handling the sim2real gap with accompanying strengths and limitations. Ultimately, the method choice largely depends on various factors: available model, input parameters/variables, investigated movement and of course the underlying research aim. Researchers should be aware that the sim2real gap remains for both ID and FD approaches. However, we conclude that multimodal approaches tracking kinematic and dynamic measurements may be one possible solution to handle the sim2real gap as methods tracking multimodal measurements (some combination of sensor position/orientation or EMG measurements), consistently lead to better tracking performances. Initial analyses show that motion analysis performance can be enhanced by using multimodal measurements as different sensor technologies can compensate each other’s weaknesses.

## 1 Introduction

In the virtual world, individuals are depicted by digital human models. The models can be used to simulate human properties and skills (Bullinger-Hoffmann and Mühlstedt, 2016). Musculoskeletal models are digital human models used to determine a person’s internal dynamic state. They depict the human body as a multi-body system and consist of rigid bodies, joints and muscles. The models can be used to compute biomechanical parameters like muscle forces, joint torques and joint reaction forces non-invasively. Musculoskeletal simulations can address research questions that are difficult to address (well) with direct measurements, either due to the definition of the variable of interest (such as center of mass) or ethical reasons. Musculoskeletal simulations have been used in the medical context to simulate the effect of muscle or tendon surgeries (Herrmann and Delp, 1999) or to investigate abnormal gait (Arnold et al., 2005; Higginson et al., 2006; John et al., 2013). The simulations have become more prevalent in various fields, including product development and human factors engineering. For example, they have been used to aid in the development of exoskeletons (Ferrati et al., 2013; Uchida et al., 2016; Molz et al., 2022), optimize worker movement at workstations (Maurice et al., 2019), and enhance the ergonomic design of products (Rasmussen et al., 2012; Jeang et al., 2018).

The general differential equation of motion is the basis for dynamics simulations of human motion

$$M\left(q\right)\ddot{q}+C\left(q,\dot{q}\right)\dot{q}+G\left(q\right)-A\tau -\;J^{T}\left(q\right)F_{ext}=0$$

where $q,\dot{q}$ and $\ddot{q}$ describe the generalized coordinates, velocities and accelerations, respectively. For the most part, generalized coordinates describe joint angles which depict the pose of the human. Additional generalized coordinates define the position and orientation of the model’s root segment (usually the trunk or pelvis) in space. M is the mass/inertia matrix dependent on the joint angles. C denotes the centrifugal/coriolis matrix, dependent on joint angles and velocities. G is the gravity matrix, dependent on the joint angles. $J^{T}\left(q\right)F_{ext}$ describes external forces using the Jacobian. τ describes joint moments and A is the coefficient matrix that converts joint torques to segment torques.

The general differential equation of motion can be formulated in two ways. It can either solve the equations of motion for the joint moments, called inverse dynamics (ID). For this, the generalized coordinates and external forces are needed as input. Or it can solve the equations of motion for the generalized accelerations using joint torques or muscle activations and external forces as input, called forward dynamics (FD). ID is part of the conventional musculoskeletal simulation workflow consisting of inverse kinematics (IK), ID and static optimization. IK computes the motion of the model, expressed in generalized coordinates, based on experimental measurements of body motion. ID uses the output of IK and measured external forces (e.g., ground reaction forces, GRFs) to compute corresponding joint loads. Static optimization computes muscle activations and muscle forces based on the output of ID. FD uses joint torque trajectories as input to generate body motions. Alternatively, muscle activation patterns, which in turn result in joint torques by using muscle models, can be used as input. An FD simulation problem can be solved using an open-loop optimal control problem also known as trajectory optimization (Chao and Rim, 1973; Anderson and Pandy, 2001).

However, since simulations are always simplifications of reality, errors are present in the outputs of ID and FD, which display in different ways. In ID, these errors show up as residual forces and torques (residuals). These residuals show up in the equations of motion for the generalized coordinates for which no input is available, specifically the degrees of freedom that define the position and orientation of the reference segment, typically the trunk, in space. Residuals occur because the model is expected to perform motions and produce forces that it did not perform and cannot produce by definition. This is because the dynamic model of the model is fundamentally different from the dynamics of the real system (the human). Hatze (2002) called this the fundamental problem of musculoskeletal simulations. External and inertial forces and torques are not in balance. Residuals are needed to dynamically balance the simulation (Reinbolt et al., 2011; Hicks et al., 2015) to ensure that the kinematic trajectory of the system’s center of mass is physically consistent with experimentally measured GRFs (Werling et al., 2023). In contrast, such residuals are not present in FD, since the dynamics of the musculoskeletal model are followed by definition. Instead, the joint angles will be different to those calculated with IK, and have a larger deviation from the measured data. IK mostly uses optimizations to calculate joint angles by finding the best match between experimental marker positions and virtual markers placed on the model for every time step. This is then called kinematic tracking, as the model tracks the experimental data.

This review focuses on musculoskeletal simulations which analyze/track experimental data. Pure FD simulations not considering experimental data, or using experimental data only for generating an initial guess, are excluded. This article focuses on the analysis of measured motion rather than producing predictions of motion, which is the focus of these simulations.

To achieve reliable simulation results, a model that is as well adapted as possible to the individual person is a prerequisite. Model individualization methods are used to adjust a generic musculoskeletal model to best fit measurements (e.g., marker positions in static pose or segment lengths) taken from the person. Despite this individualization process, deviations between reality and the simulation will remain. We call this deviation the sim2real gap.

The sim2real gap has two error components: the kinematic and the dynamic error. The kinematic error is the difference between joint angles of the real human and the model. As the true joint angles are usually not measurable, the kinematic error can be expressed by the sum of squared differences between measured and simulated marker trajectories or sensor positions/orientations. Kinematic errors stem from experimental errors like noisy position, orientation or acceleration measurements and soft tissue artifacts (STAs). Those experimental errors are especially crucial, since the computation of joint velocity and joint acceleration through numerical differentiation leads to an amplification of those errors (Kuo, 1998). Furthermore, despite the model individualization process, there are kinematic differences between the human and the models, such as deviations of joint axes or joint rotation centers and deviating body segment dimensions. These differences also contribute to the kinematic error. The dynamic error is the difference between joint moments, muscle activity patterns and external loads of the real human and the model. As the true joint moments are not measurable, the dynamic error can be expressed by the residuals, differences in muscle activity patterns or external loads. For FD approaches, residuals cannot be observed as they are not generated. The dynamic error arises due to discrepancies between the musculoskeletal model and the human body, such as body segment inertial parameters (BSIPs) and muscle parameters, inaccurate external load measurements, and the propagation of the kinematic error. BSIPs include the mass, position of center of mass and inertia. Despite previous model individualization, deviations between BSIPs and muscle parameters still occur due to modeling assumptions or inaccurate model parameters. BSIPs and muscle parameters are not precisely known and without the use of medical imaging methods (e.g., MRI), they can only be computed using regression equations based on cadaveric measurements (Leva, 1996) or geometric approaches (Hatze, 1980).

In this review, the sim2real gap is investigated separately from model-person consistency, which describes the consistency between musculoskeletal models and their real-life counterparts. While model person consistency does influence the sim2real gap–especially because of the influence of BSIPs–integrating model individualization methods goes beyond the scope of this review.

As musculoskeletal simulation results are used to answer (patho-) physiological research questions, simulation results should be accurate and reliable. In general, four comparative values are analyzed to evaluate simulation results. For the kinematic assessment, the level of deviations between measured and estimated marker trajectories or sensor positions/orientations are analyzed. For the dynamic assessment, the size of residuals, or the deviation between measured and estimated muscle activity patterns or external load measurements can be analyzed. These comparative values can be analyzed for both ID and FD approaches. For ID approaches, Hicks et al. (2015) presented recommendations for verification and validation measures. For marker trajectories, the overall root mean square error (RMSE) should be within the measurement error. Maximum residual forces and torques are considered valid, if the residual forces are less than 5% of the magnitude of the net external force and the residual torques are less than 1% the magnitude of net external forces multiplied by the center of mass height. Relatedly, Gupta et al. (2022) presented a physics informed approach for calculating maximum residual force/torque values. They reported maximum residual value ranges that are mostly more restrictive than the recommendations given by Hicks et al. (2015). For FD approaches, no specific recommendations exist. However, if the simulation results deviate significantly from the measurement data or IK results, it is questionable whether they should then still be used to answer research questions based on measured motion data, because of the large sim2real gap.

Consequently, for accurate and reliable simulation results, solution strategies are needed to handle the sim2real gap. Begon et al. (2018) carried out a systematic review and presented existing IK methods to compensate the effect of STAs. These methods minimize the kinematic error. Publications that were included in their work are not further analyzed in our review. To handle the dynamic error in musculoskeletal simulations, different methods have been proposed. Vaughan et al. (1982) optimized BSIPs based on kinematic data and optimization theory by minimizing the difference between measured and calculated GRFs. Riemer and Hsiao-Wecksler (2009) extended this approach by optimizing both segment-angle trajectories and BSIPs to minimize the difference between measured and calculated GRFs. Thelen and Anderson (2006) implemented a proportional-differential (PD) controller to minimize residuals for walking simulations by adjusting pelvic translations and lower back angular trajectories and van den Bogert et al. (2011) used trajectory optimization to track experimentally measured motion data without generating residuals. However, no comprehensive overview of available methods exists describing how to handle the sim2real gap in musculoskeletal simulation by minimizing both kinematic and dynamic errors to achieve reliable and accurate simulation results. Therefore, we conducted a systematic literature search to identify, classify and analyze existing methods that handle the sim2real gap in musculoskeletal simulations. Our research questions are:

RQ1: Which solution approaches exist to handle the sim2real gap in the field of musculoskeletal simulations?

We give readers an overview of available methods using both the ID and FD simulation approach that can be used to handle the sim2real gap in musculoskeletal simulations.

RQ2: What is the primary goal of the identified methods and which strengths and limitations do they have?

We describe strengths and limitations of the methods to help researchers decide which approach is most appropriate for their particular research question. In addition, we analyze if methods exist that are able to handle the sim2real gap for any arbitrary model, input parameters and investigated movements without deviation from experimental data.

This review is intended for both beginner and more experienced researchers in the field of biomechanical simulations. Beginners who are just starting out with biomechanical modeling and simulations are given a comprehensive explanation of the sim2real gap as well as an overview of possible simulation methods that are generally used in this field. More experienced researchers who are concerned about the sim2real gap of their simulation results can use the overview of identified methods to decide which method would be best for answering their individual research question.

## 2 Survey methodology

This review was planned following the PRISMA-P 2015 checklist (Moher et al., 2015). We followed a systematic approach to identify relevant literature. An electronic search was performed in the databases Scopus, PubMed and Web of Science (cut-off date: 24 January 2024), followed by a manual screening and selection process. Results were first screened by title and abstract. After that a full-text screening was performed to determine eligibility. Documents of the types *article* and *review* in English language were considered. Further limitations were chosen for every database based on available limitation criteria. For Scopus, the search string was applied to title, abstract and keywords. The results were limited to the following search categories: Engineering, Medicine, Computer Science, Mathematics, Physics and Astronomy, Multidisciplinary. For Web of Science, the search was performed on all fields for the Web of Science Core Collection. No further limitations were chosen. For PubMed, the search was performed only on title and abstract as there was no option to search keywords. As our goal was to identify methods that handle the sim2real gap, we applied a comprehensive search string:

(("musculoskel*") OR ("musculo-skel*") OR ("biomechan*") OR ("anthropo*") OR ("digital human")) AND (("model*")) AND (("kinemat*") OR ("dynam*") OR (" kinet*")) AND (("method*") OR ("approach*") OR ("framework*") OR (" algorithm*")) AND (("motion*") OR ("gait*") OR ("movement*")) AND (("error*") OR ("inconsisten*") OR ("miscalcu*") OR ("minim*") OR ("consisten*") OR ("incorrect*")) AND (("transfer*") OR ("track*") OR ("reconstruct*") OR ("optim*") OR ("simulation*") OR ("residual*"))

Search items included relevant terms like kinematic, dynamic, residuals, consistency and errors. We used a generic search string since the term *sim2real gap* is not an established term in the field of biomechanical simulations. Additionally, a backward search identifying further works was performed on identified relevant publications.

Results retrieved by the search string were appraised for significance according to specific inclusion and exclusion criteria, which were applied first to the title, then to the abstract, and then to the full-text. Papers describing methods handling the sim2real gap of musculoskeletal simulations using a physiological biomechanical model were included. These methods should compensate (or minimize) either the kinematic error, the dynamic error or both errors simultaneously. Papers describing model individualization methods as well as pure FD simulations that do not track experimental data are out of scope for this research and therefore excluded as this review focuses on handling the sim2real gap in musculoskeletal simulations to achieve accurate and reliable simulation results based on experimentally measured motion data. One author screened all records and decided whether a publication met the inclusion criteria. If the author was unsure whether or not to include the work, he consulted his co-authors. In the end, one other author checked all identified publications for eligibility.

To answer the aforementioned research questions the papers were analyzed according to the following aspects: problem formulation, input/output, model specifics, method evaluation, simulation software, states/controls, experimentally measured quantities and the primary goal of each method. No automation tools were used for this process. For every study, each aspect was analyzed and written in a spreadsheet. One author extracted the data. A second author checked the accuracy of the extracted data and the completeness of the spreadsheet.

To synthesise the data, the analyzed aspects were used to cluster the identified studies according to the way how the sim2real gap is handled, the mathematical method used, and the parameters/variables of the simulations which were adjusted which corresponds to RQ1.

## 3 Results

### 3.1 Search yield

**FIGURE 1:**
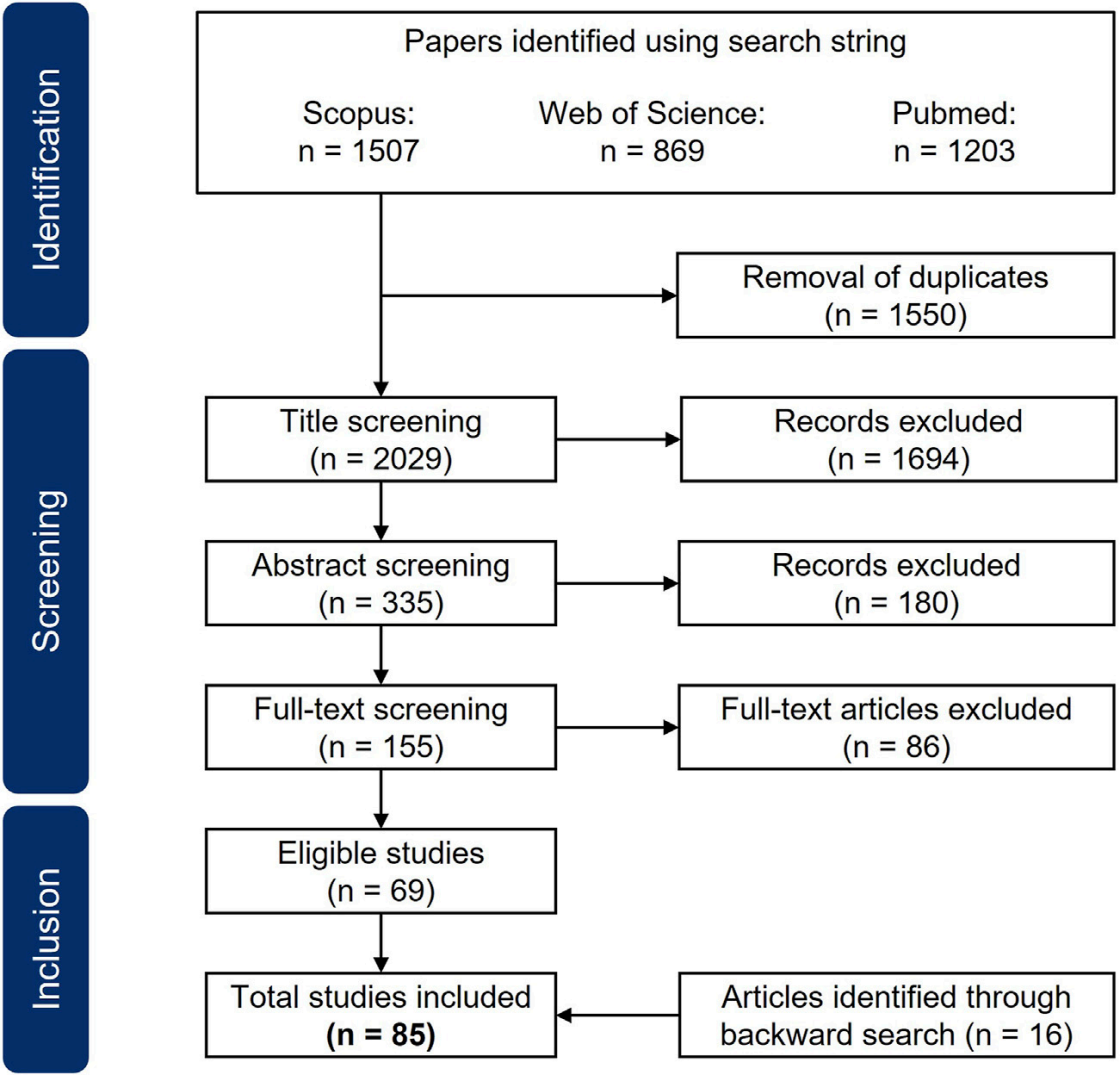
PRISMA flow diagram describing the literature search, identification, screening and selection process. The search string was applied to the three databases Scopus, Web of Science and Pubmed. It yielded 3579 results. The results were first screened by title, then by abstract and then the full-text was screened for eligibility using defined inclusion criteria. Backward search was performed on eligible results. In the end 85 papers were included in the review.

Applying the search string to the three databases yielded 3,579 results. Figure 1 illustrates the identification and selection process. We used the aforementioned inclusion and exclusion criteria to exclude non-relevant publications. 1,550 duplicates were removed. After screening the results by title, 335 results remained. The abstract screening excluded a further 180 papers. In the end, 86 papers were excluded through full-text screening. In turn, 16 publications were added by performing backward search. Ultimately, 85 results fulfilled the criteria and thus were included in this review.

### 3.2 Identified solution approaches (RQ1)

**FIGURE 2:**
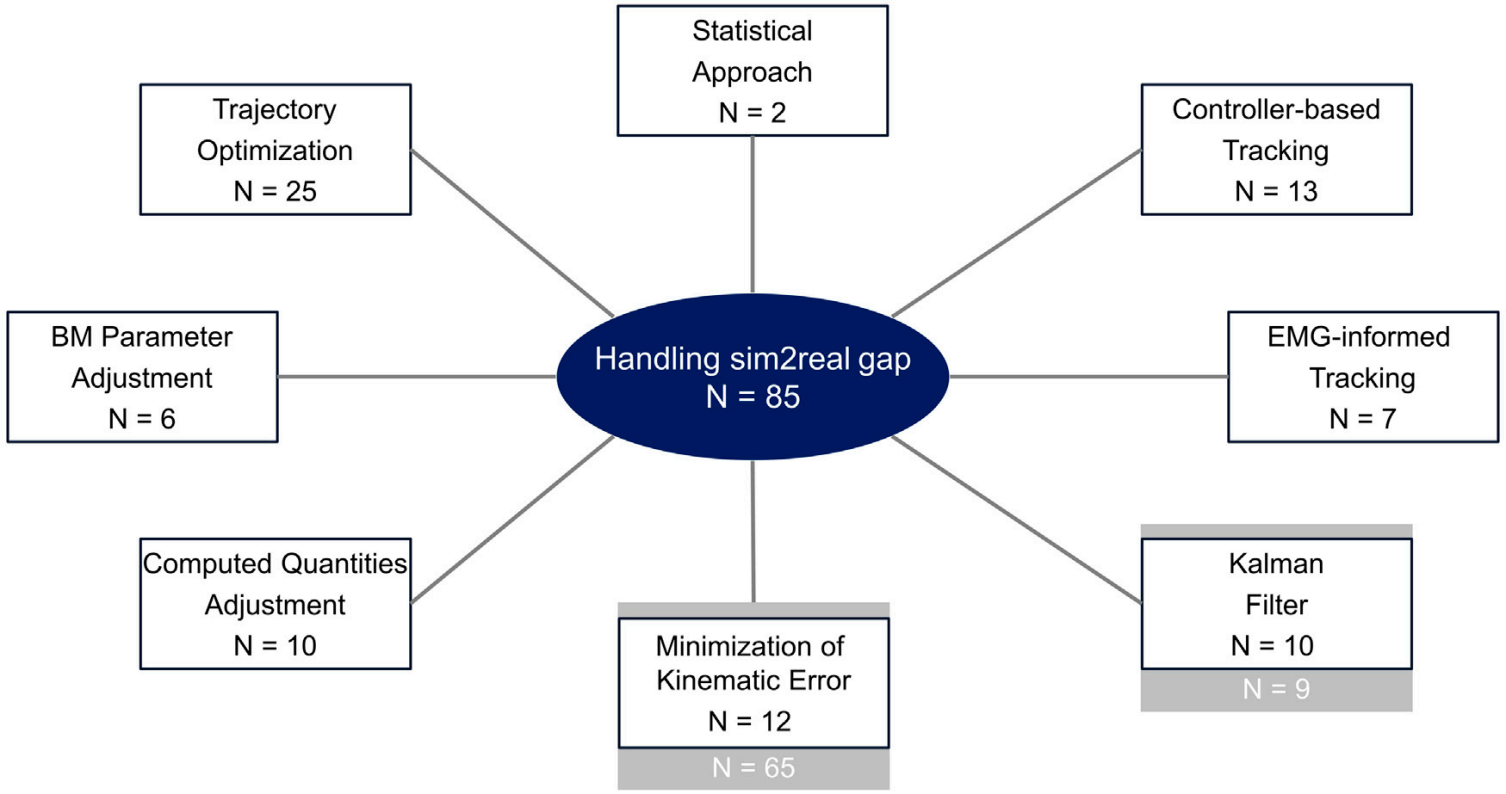
Relevant identified publications were sorted into eight different solution approaches (clusters). The approaches differ in three aspects: how the sim2real gap is handled, the mathematical method used and the parameters/ variables of the simulations which were adjusted. The grey boxes represent publications that were already analyzed by Begon et al. (2018) in their literature review regarding inverse kinematics methods. As these methods aim to minimize the kinematic error they also handle the sim2real gap.

We clustered relevant publications into eight general solution approaches (clusters), see Figure 2. These approaches differ in the way the sim2real gap is handled, which mathematical method is used and which parameters/variables of the musculoskeletal model or the simulation are adjusted to handle the sim2real gap. Table A1 in the Supplementary Material shows the cluster to which each publication is assigned. Each cluster is briefly described in terms of how the sim2real gap is handled. Furthermore, Table A2–Table A9 summarize, separately for each cluster, the proposed method. These tables include additional information for each cluster, including if and how the method was validated. If a marker-based motion tracking approach was used as reference, we stated that the method was validated using the standard approach. If the method was verified on synthetic data, we also indicated that. If it was not validated, we added information regarding the performance (e.g., kinematic RMSE) of the proposed method or information regarding the evaluation (e.g., residual size, comparison between measured and computed GRFs) in the corresponding column. Table A10 in the Supplementary Material lists experimental measurements that were used in every publication.

Methods in the *minimization of kinematic error* cluster describe kinematic tracking methods that handle the sim2real gap by optimizing kinematic tracking performance. Based on the kinematic input being tracked, the methods either compensate noise and sensor drift (inertial measurement unit (IMU) -based methods), STAs (marker-based methods) or enhance the tracking performance by including joint constraints (depth camera-based methods). Method descriptions and information about kinematic inputs are listed in Table A2.

Methods included in the *BM parameter adjustment* cluster principally use the fact that the ID problem is overdetermined when both external forces and kinematics are measured. In this case, the sim2real gap is handled through optimization, where either BSIPs or marker positions are adjusted to reduce residuals. Method descriptions and information about optimized variables are listed in Table A3.

Methods included in the *computed quantities adjustment* cluster, generally use a similar approach like those of the *BM parameter adjustment* cluster. These methods also take advantage of the overdetermined nature of the ID approach to handle the sim2real gap. The difference between the two clusters are the input variables of the cost functions which are modified. Instead of optimizing BSIPs or marker positions, computed variables like joint angles, joint accelerations or muscle forces are adjusted. Method descriptions and information about optimized variables are listed in Table A4.

Another solution approach (cluster) is the implementation of a *Kalman Filter* to handle the sim2real gap for musculoskeletal simulations. In general, the Kalman Filter is a two-step prediction correction approach generating optimal state estimations based on erroneous measurements (Serra, 2018). It produces optimal estimates of a system state by averaging predicted and measured states using a weighted average. The covariance of both the system and the measurement noise is used to calculate the weight. Through this, random noise of measurement systems can be reduced. In case of biomechanical simulations, the state vector of the Kalman filter may include only kinematic parameters (e.g., joint angles). These approaches then minimize the kinematic error. The state vector can also include both kinematic and body segment parameters (Bonnet et al., 2017) or both kinematic and kinetic parameters (e.g., joint angles and muscle forces or joint torques) (Mohammadi et al., 2020). Or it can include uncertainty models of IMU-sensors to estimate the size of undesired errors (orientation errors, gyroscope bias and magnetic disturbances) to compensate these (Yuan et al., 2019). The choice of the state vector variables mainly depends on the underlying study aim–minimization of the kinematic error or dynamic error. Method descriptions and state vector variables for every publication classified into this cluster are listed in Table A5.

Methods in the *trajectory optimization* cluster use both the FD and ID approach to generate biomechanical simulations. FD approaches generate simulations without residual forces and torques. For approaches using ID the dynamical constraints are loosened and residuals are set to some level of tolerance. In contrast to static optimization, trajectory optimization mostly considers muscle activation and deactivation dynamics and optimizes the movement over the whole time series. This way non-physiological jumps in position and acceleration data are prevented. This aspect applies for all papers included in this cluster. A subcategory of this cluster is called inverse dynamic optimal control. Inverse dynamic optimal control uses trajectory optimization to solve the muscle redundancy problem dynamically over time. It computes neural excitations that result in given joint torques, which were computed using ID. Thus, the methods produce motion simulations that are consistent with muscle dynamics but not necessarily consistent for the external forces. Table A6 lists method descriptions as well as information about the optimization variables as these differ.

Methods in the *electromyography (EMG)-informed tracking* cluster are multimodal motion tracking methods. They use cost functions which are extended by including EMG-measurements, as an expression of neural excitations, in addition to marker or joint angle trajectory tracking. All methods use trajectory optimization to solve a forward dynamic simulation problem to dynamically track both measurements. It is assumed that tracking both marker trajectories and EMG-measurements enhances accuracy and reliability of biomechanical simulation results (Bailly et al., 2021). Table A7 lists method descriptions as well as information about the optimization variables.

Methods in the *controller-based tracking* cluster use the FD approach. They handle the sim2real gap by applying a dynamic tracking approach. Next to kinematic parameters also dynamic parameters (e.g., muscle activations) are being tracked. However, a unique feature here is the implementation of feedback into musculoskeletal simulations. Additionally, some kind of controller is used to track specific input variables. The methods use the FD approach. This way no undesired residuals are generated. Most methods included in this cluster focus on enhancing accuracy of simulations in the field of gait analysis. Some methods focus on enhancing accuracy of simulations for wrist or arm movement. Table A8 lists method descriptions and information about the controller that was used.

The last cluster includes methods using *statistical approaches* for musculoskeletal simulations and to handle the sim2real gap. Instead of minimizing specific cost functions, as was the goal in all other clusters, statistical approaches try to estimate the most likely variable values (e.g., joint angles or joint loads) given a specific set of measured kinematic parameters (e.g., marker trajectories). Method descriptions and information regarding the statistical approach are listed in Table A9.

### 3.3 Primary goal in relation to handling the sim2real gap, strengths, and limitations of methods (RQ2)

The primary goal varies for all listed publications and methods. Some methods aim at tracking kinematic parameters as closely as possible and thus minimizing the kinematic error (Cesic et al., 2016; Begon et al., 2017; Bonnet et al., 2017; Joukov et al., 2017; Laidig et al., 2017; Schellenberg et al., 2017; Joukov et al., 2018; Niu et al., 2018; Tagliapietra et al., 2018; Pataky et al., 2019; Inai et al., 2020; Joukov et al., 2020; Price et al., 2020; Sy et al., 2020; Halilaj et al., 2021; Sy et al., 2021; Al Borno et al., 2022; Lefebvre et al., 2023). Alternatively, the kinematic error can be minimized by compensating sensor noise and bias (e.g., sensor drift or magnetic disturbances) which affects methods using IMU-sensors for motion analysis (Cockcroft et al., 2014; Allen et al., 2017; Laidig et al., 2017). Some methods investigate if a multimodal motion analysis approach (combination of IMU-sensors with either RGB-video- or depth-camera) enhances tracking performance thus minimizing the kinematic error (Atrsaei et al., 2016; Halilaj et al., 2021; Pearl et al., 2023). Other methods explicitly aim to generate simulation results without undesired residuals (Vaughan et al., 1982; Koopman et al., 1995; Kuo, 1998; Cahouët et al., 2002; Mazzà and Cappozzo, 2004; Riemer et al., 2008; Remy and Thelen, 2009; Riemer and Hsiao-Wecksler, 2009; Jackson et al., 2015; Samaan et al., 2016; Faber et al., 2018; Muller et al., 2018; Noamani et al., 2018; Fritz et al., 2019; Pallarès-López et al., 2019; Sturdy et al., 2022; Werling et al., 2023). Certain methods primarily target a different goal than generating simulation results without residuals. The goal can be the estimation of most accurate muscle forces or torques (Chao and Rim, 1973; Koh and Jennings, 2003; Seth and Pandy, 2007; Morrow et al., 2014; Lv et al., 2016; Bélaise et al., 2018a; Bélaise et al., 2018b; Stanev and Moustakas, 2018; Moissenet et al., 2019; Bailly et al., 2021; Ceglia et al., 2023), the determination of joint torques or muscle excitations needed to generate desired movement (Kaplan and Heegaard, 2001; Thelen et al., 2003; Thelen and Anderson, 2006; Da Silva et al., 2008; Ghafari et al., 2009; Demircan et al., 2010; Watanabe and Sugi, 2010; Lin et al., 2018; Mouzo et al., 2018; Dorschky et al., 2019; Haralabidis et al., 2021; Wang et al., 2021; Wang et al., 2022) or the development of controllers to determine muscle activity that track desired movement (Yamaguchi and Zajac, 1990; Blana et al., 2009; Arash Haghpanah et al., 2022). Handling the sim2real gap is then a secondary achievement. Some publications investigate the effect of different computational choices on computation time (Falisse et al., 2019) or robustness of convergence (Neptune, 1999; Febrer-Nafría et al., 2020). Others aim at predicting human motion (van den Bogert et al., 2011; van den Bogert et al., 2012; Meyer et al., 2016; Nitschke et al., 2020; Febrer-Nafría et al., 2022). Neptune and Hull (1998) developed a musculoskeletal model and optimization framework to best reproduce some specific movement and Nitschke et al. (2023) developed an approach for analyzing arbitrary three-dimensional motions using optimal control simulations by directly tracking marker trajectories and GRFs. Since the solution strategies (clusters) all differ quite extensively in how the authors (attempt to) handle the sim2real gap, all strategies have various limitations and potentials which are described below.

Methods in the *BM parameter adjustment* cluster complement the standard workflow consisting of model scaling, IK and ID as an extension but there is a risk that calculated parameters could be overfitted or unrealistic. Measures should be taken to prevent this (constraining the solution space). The methods are not limited to certain motions even though initially measurements of specific movements are required to adjust the BSIPs. The following limitations are associated with these methods. Although modeling errors are corrected, model assumptions (e.g., missing model segments) cannot be compensated. Furthermore, several assumptions are made which reduce the validity of computed results: bilateral symmetry, error-free measured kinematics and use of a correct objective function. The methods imply that all residuals stem only from inaccurate BSIPs. This assumption leads to the estimation of values that minimize a specific cost function but do not have to be equal to the real value. Muller et al. (2017) even state that an optimization of BSIPs based on the difference between calculated and measured GRFs is not necessary as the risk of overfitting cannot be avoided.

The work of Werling et al. (2023) is an exception to the points previously discussed. The paper presents *AddBiomechanics,* an online tool that enables the computation of motion dynamics analysis in an automated and standardized way. Marker trajectories are tracked as close as possible by optimizing joint angles, body segment parameters as well as model marker positions simultaneously. Additionally, the model’s center of mass and rotational generalized coordinates of the model’s root segment are adjusted to be consistent with experimentally measured GRFs. Therefore, the residuals are not assumed to be solely generated by either erroneous kinematics or BSIPs. Variables of both entities are adjusted to handle the sim2real gap without deviating too much from the kinematic input data. Nevertheless, there are still limitations. A statistical prior model is used to optimize the body segments. This prior model is based on population data of active-duty military personnel (men and women) and is therefore not reflective of the broad population. The authors state that the method may then choose body segment parameters for fitting to the marker data that are more in line to the parameters in the prior model, even though the “true” parameters are different. Additionally, the optimizer prioritizes solutions that move anatomical markers as little as possible. If the experimental markers have not been labelled correctly, this could lead to the estimation of incorrect joint angles and the model marker positions on the input model would have to be adjusted accordingly to compensate this.

Strengths and limitations of methods in the *computed quantities adjustment* cluster are similar to aspects described in the previous paragraph. The methods significantly minimize residuals but a risk of overfitting or computing unrealistic parameter values remains. One strength is that it is possible to constrain optimization parameters into physiologically reasonable ranges, as done by Riemer and Hsiao-Wecksler (2009), to prevent the estimation of unrealistic parameter values. Computed parameters are assumed to be inaccurate due to measurement noise and STAs. Different kind of errors can be compensated depending on the described method. Cahouët et al. (2002) compensate derivation errors in joint accelerations. Koopman et al. (1995) compensate errors stemming from both measurement and model segment estimation errors, whereas Kuo (1998) compensates the effect of noise on position data. While this does reduce the size of residuals in the simulation, the compensation is done by deviating from the experimental data. The assumptions made in these methods, however, limit the validity of the computed results. In most publications BSIPs and GRFs are assumed to be error-free and it is assumed that residuals stem only from inaccurate kinematic parameters. Accordingly, risk of overfitting towards the model cannot be avoided. Bonnet et al. (2017) explicitly state that unrealistic accelerations may be generated since an optimization algorithm compensates for various error types and not only for differentiation errors as intended in the work of Cahouët et al. (2002) and Kuo (1998). In addition, some methods were developed for analyzing only one specific movement and thus are not universally applicable.

Using *trajectory optimization* to determine biomechanical parameters can lead to simulation results without residuals. However, to achieve this, simulation results will move away from marker trajectories, IMU sensor measurements or IK results. In general, the cluster has a couple of strengths. The variables are optimized dynamically over time. This way, non-physiological joint angle, velocity and acceleration trajectories are avoided because muscle activation/deactivation dynamics are generally implemented. Methods in this cluster may generate simulation results without residuals as trajectory optimization is one possible way to solve FD and the simulations adhere to the laws of physics. Moreover, the methods are flexible. States, controls, constraints as well as the objective function can be chosen to reach a specific goal. Cost functions can be extended to not only track kinematic but also kinetic parameters (e.g., GRFs) to enhance convergence performance. If a foot-ground contact model is added, GRFs can be computed and compared to measurements. Thus, force plate measuring errors and modeling assumptions can be compensated through the adjustment of GRFs. The limitations for this solution approach include that measured motion data may be smoothed significantly in order to adhere to system dynamics. A further limitation is the high computation time and the amount of computational power that is required for solving forward dynamic formulations. The simulations may have problems to converge. This may be especially true for non-cyclic movements, as periodicity constraints can then not be applied. Furthermore, choosing optimal weights for the different terms of the objective function (tracking term, regularization term, smoothing term) is challenging. Additionally, trajectory optimization methods assume that the model BSIPs are accurate and do not get adjusted, only the system kinematics and forces are optimized. Applying trajectory optimization on three-dimensional models with many degrees of freedom and muscles significantly increases the complexity and therefore computation time increases and convergence becomes even more challenging. Error propagation occurs when joint angle trajectories are directly tracked, because the reference joint angle trajectories result from an IK which is error-prone. These erroneous joint angle trajectories are then used as reference for the simulation.

Using a *Kalman Filter* minimizes the kinematic error of simulation results as error models are used to compute optimized state estimations (e.g., marker positions, sensor positions or orientations). However, the quality of the optimized state vector estimations is restricted by the accuracy of the implemented error models. The *Kalman Filter* offers unique advantages when applied for musculoskeletal modeling and simulation since the method was specifically developed to enhance state estimations (originally position location) based on erroneous observations (Serra, 2018). Mohammadi et al. (2020) produced estimates that are both consistent with the system and the measurement model. They assumed that joint angle measurements are affected by white Gaussian noise; e.g., modelled as a normal probability distribution. This noise can then be compensated by the extended Kalman Filter because the explicit noise model is known. Analogous to the computed quantities adjustment cluster, state variables can be constrained to ensure feasible parameter values (Bonnet et al., 2017). Nevertheless, a Kalman Filter has many parameters that need to be set correctly, requiring expert knowledge. Covariances have to be known to compensate measuring noise and STAs cannot yet be compensated in contrast to white gaussian measurement noise since no coherent error model exists. Further, the Kalman Filter is currently mostly applied for simulating two-dimensional models (Bonnet et al., 2017; Mohammadi et al., 2020). Bonnet et al. (2017) state that, although possible, expanding their method to include three-dimensional model estimation would require significantly more parameters and could lead to redundancy problems. Moreover, for many publications, specific assumptions are taken to enhance or enable convergence: bilateral symmetry, constant acceleration or jerk, a fixed yaw angle or periodicity in gait. This significantly reduces the validity of presented methods.

Since all reviewed *EMG-informed tracking* methods use trajectory optimization as mathematical method, strengths and limitations are analogous to previously described aspects. Besides those aspects, it is advisable to track both EMG-measurements and marker trajectories because the measurements complement each other (Bélaise et al., 2018b) and minimize both the kinematic and dynamic error simultaneously. However, EMG-measurements are error prone and thus may restrict the significance for handling the sim2real gap. The methods enable the implementation of muscle co-contraction in musculoskeletal simulations in contrast to using cost functions minimizing the sum of squared muscle activity. Furthermore, overall muscle force estimation can be enhanced (Bailly et al., 2021; Ceglia et al., 2023). Main limitations for methods of this approach are challenges related to EMG measurements, which are often noisy, while physiological cross-talk cannot be avoided. Also, surface electrodes cannot be used to measure activity in deep muscles (Sartori et al., 2014). In addition, external disturbances may interfere with the measurements and lead to inaccurate values.

*Controller-based tracking* methods can generate simulation results without residuals but may move away from measured parameters to achieve this (Thelen and Anderson, 2006). The methods tend to not only model the human body, but also the control of the human nervous system by incorporating feedback in the simulation. Moreover, analogous to the *trajectory optimization* cluster, kinematic parameter trajectories (e.g., joint angles) remain physiologically feasible because muscle activation and deactivation mechanics are implemented. Controller-based tracking approaches may have problems to accurately track movements that are characterized by rapid acceleration changes (e.g., jumps or running) if the feedback gains are not implemented correctly (van der Kooij et al., 2001; Koelewijn and Ijspeert, 2020). Using sensory input, the healthy human body is able to react incredibly fast to disturbances like sudden imbalance in form of tripping while walking. In this case, the postural controller reacts immediately and tries to prevent the person from falling. While some controllers are somewhat/partly able to imitate measurements of human behavior after perturbations, it is not known if the model that is used (e.g., reflex controller or a PD controller) is analogous to the control as it happens in the human body. In addition, typical limitations which are associated with controllers can also occur, including instability and loss of control.

*Statistical approaches* minimize the kinematic error by maximizing a specific probability (e.g., maximum likelihood of a parameter’s posterior distribution) instead of minimizing specific objective functions which leads to more accurate joint angle estimates in contrast to standard approaches (Pataky et al., 2019). However, the methods are restricted by convergence problems and the required high computational effort. Lv et al. (2016) generated a data-driven prior-model which uses known information from a motion database to constrain the solution space of the inverse dynamic problem. This way, ambiguity of human motion (e.g., during double stance phase of gait) gets reduced, which occurs when there is no information available regarding the distribution of the ground reaction forces between both legs. Generally, limitations for these approaches include that analogous to the Kalman Filter cluster, convergence problems may occur when implementing three-dimensional models. Pataky et al. (2019) introduced the Bayesian IK method and state that it is impracticable for more complex models with many degrees of freedom. Furthermore, significant computational power is needed and computation time is already high for two-dimensional analyses (Pataky et al., 2019). Moreover, since the method of Lv et al. (2016) is based on a database including specific motions, only the included motion types (walking, jumping, running, turning and hopping) can be accurately analyzed. Results for other movements (e.g., stair climbing) were not accurate. A generalization to related but nevertheless different movements is therefore not possible.

## 4 Discussion

The literature study revealed answers to the raised research questions which are summarised next.

RQ1: For the first research question, we investigated which methods exist to handle the sim2real gap in musculoskeletal simulations and found that the results could be classified into eight clusters: *minimization of kinematic error*, *BM-parameter adjustment, computed quantity adjustment, Kalman Filter, EMG-informed tracking, controller-based tracking, trajectory optimization* and *statistical approaches.* The clusters differ in the way the sim2real gap is handled.
RQ2: For the second research question, we investigated the primary goal of the methods regarding the way the sim2real gap is handled as well as strengths and limitations of each cluster. In short, every method has a distinctive primary goal. Every cluster has a distinctive way how to deal with the sim2real gap with accompanying potentials and limitations. We could not identify a specific solution approach that is able to generate consistent simulations without introducing residuals for any arbitrary model, input variables and investigated movement without deviating from the corresponding experimental data. At this point, the automated tool AddBiomechanics presented by Werling et al. (2023) seems to be the best solution for analyzing experimental kinematic and kinetic data using ID, as it optimizes both kinematic and kinetic input and estimated parameters to handle the sim2real gap. But the method is only able to analyze marker-based motion data. For FD, methods in the *EMG-informed tracking* cluster are very promising, as these methods handle the sim2real gap by minimizing both the kinematic and dynamic error simultaneously.

The majority of the reviewed methods was designed for specific movements like human gait (e.g., Koopman et al., 1995; Thelen and Anderson, 2006; Remy and Thelen, 2009). Thus, most of the methods are not suitable for handling the sim2real gap for arbitrary movements. Although some papers offer promising methods (e.g., Kalman Filter, Bayesian IK), the majority of these are implemented for two-dimensional models. Application to three-dimensional models would require considerably more computational power. Convergence and redundancy problems could also occur (Bonnet et al., 2017; Pataky et al., 2019). Some methods focus solely on one of the reasons contributing to the sim2real gap mentioned in the introduction (e.g., measurement noise, STAs or modeling errors). This mainly includes publications from the *BM parameter adjustment* and *computed quantities adjustment* clusters. These solution approaches are problematic since residuals describe how large the dynamic error of the sim2real gap is. Often, in an optimization, it is assumed that all error is due to either the measurements or the model. The assumed source is adapted to reduce or eliminate the residuals. However, this adaptation then includes the error from the other source. Consequently, in case of musculoskeletal simulations, parameter values (e.g., BSIP, joint angle, joint acceleration trajectories) which are generated by such an approach do not have to correspond to the real values. For methods included in these clusters, there is high risk of overfitting optimization parameters. In addition, the dynamic error of the simulation may be decreased after adjusting specific values to minimize residuals, but this does not necessarily enhance the validity of the simulation. In fact, the consistency between the BM and the corresponding person can even decrease. As a result, the sim2real gap increases and thus the reliability of the simulation decreases. One way to see this, is by analyzing an independent experimental measure, e.g., muscle activity, and compare this to the corresponding model output. Furthermore, in order to gain valid simulation results without residuals with these approaches, it should be known which part of residuals stems from which error (modeling or measurement error). As there is no way to determine this distribution it seems advisable to generate a model that represents a specific person as accurately as possible before trying to reduce residuals with other solution approaches. Methods using trajectory optimization generate simulation results without residuals by definition but may significantly move away from experimental data as model parameters do not get adjusted during the optimizations. This may be less relevant if the overall aim of the investigation is to predict either novel movements or to analyze the change biomechanical variables due to changing environment or product design (e.g., for the design of running shoes as in van den Bogert et al. (2012)). However, when analyzing experimental data, this approach may lead to solutions which are not reliable or accurate for the person initially measured. This aspect mainly depends on the chosen term weights of the optimization problem. A higher tracking weight can be chosen to force the simulation to track the input data more closely but then convergence problems may arise as the solver is not able to find a solution. Analogous, c*ontroller-based tracking* methods also generate simulation results without residuals and may move away from experimental measurement data so that the simulation adhere to system dynamics. However, the methods may be advantageous for tracking non-periodic data as no periodic constraints are defined to enhance convergence. *EMG-informed tracking* methods track both kinematic and dynamic variables, decreasing the kinematic and dynamic error simultaneously. Through this multimodal approach, the sim2real gap is handled in a more holistic approach.

Currently, there is no universal strategy to handle the sim2real gap for an arbitrary movement while using an arbitrary model and arbitrary motion data because the problem is extensive and complex. This is also emphasized by the diverse solution approaches (clusters) which were identified in this review. There is no consensus on which method is most appropriate for achieving reliable and accurate simulation results. Hicks et al. (2015) provide recommendations for reducing and minimizing residuals in case the ID approach is used. This includes carefully executing pre-processing steps. Data collection should be well prepared and the calibration procedure should be performed diligently. Furthermore, the model should be scaled properly. If residuals are still large, parameters like inertia values or kinematics can be adjusted. These recommendations are in accordance to clusters identified in this review (*BM parameter adjustment* and *computed quantities adjustment*) and to suggestions that Hatze proposed in his work in (2002) in which he described the fundamental problem of myoskeletal inverse dynamics. He implied that sufficiently complex musculoskeletal models as well as error reduction in data measuring and processing methods are necessary to improve the accuracy of computed joint torques using ID. Analogous, Riemer et al. (2008), Riemer and Hsiao-Wecksler (2009) and Gupta et al. (2022) show that residuals have an effect on computed joint torques. In case a full-body model is used for an analysis, researchers have to take care that their residuals are not larger than values that are recommended by Gupta et al. (2022). Otherwise, the computed joint torques are neither accurate nor reliable (and should not be taken as a basis for answering (patho-) physiological questions). The online tool *AddBiomechanics,* presented by Werling et al. (2023) seems to be the most sophisticated and also holistic approach to handle the sim2real gap using the ID approach. As both kinematic and kinetic input and estimated parameters are optimized to achieve minimum residuals, this framework is consistent with the solution strategies identified in this review.

However, the aforementioned proposed recommendations apply only for ID approaches. For FD approaches used to analyze experimentally measured motion data, no specific recommendations are given in the literature on how to handle the sim2real gap. In this review this includes the *trajectory optimization*, *controller-based tracking* and *EMG-informed tracking* clusters. FD approaches generate no residuals by definition but to achieve this, the simulation may move far away from experimental measurements or IK results and the larger the sim2real gap again becomes. Researchers using FD to analyze experimental measurement data should keep this in mind if they plan to use the simulation results to answer (patho-) physiological research questions.

At this point it should be mentioned that achieving simulation results without generating residuals is not necessary or should not be tried to achieve in any case. If there was a theoretically perfect model of a person available the remaining inconsistency would stem solely from the measurement errors and could then be eliminated using a FD approach. Since perfect, fully comprehensive models are not possible to achieve, each model is only an abstraction depicting a real person in more or less detail. Based on the idea that a simulation conducted with a perfect model generates no modeling errors, one can argue that the more accurate the model is, the fewer modeling errors are expected. Greater modeling errors would be assumed when using partial models in comparison to using full-body models. For this reason, it is not appropriate to strive for handling or closing the sim2real gap when a partial model of the human body is used. Researchers have to take care how much they should strive for minimizing the kinematic and dynamic error given the accuracy of the model they use. The less complex a model is, the less clear it will be which part of the residuals is generated by each error source (modeling or measurement error). Minimization of residuals is then not advisable. On the contrary, van den Bogert and Su (2008) state that in this case, and on the condition that full ground reaction force measurements are available, more reliable ID results can be generated when the upper-body is not included because its motion cannot be measured reliably and minimizing residuals would thus introduce errors in the simulation results.

We described in the previous paragraph that removing residuals is not recommended for any model. In these cases, the size of residuals can also not be used to validate a musculoskeletal simulation as proposed by Hicks et al. (2015) and Gupta et al. (2022). Validating musculoskeletal simulation results remains a challenge because the ground truth can never be known. As optical marker tracking is the gold standard for motion capture, new measurement and simulation methods are validated using this approach. Nevertheless, the gold standard is also prone to both modeling errors and measurement noise. When a new method produces results that differ from the gold standard solution, it is hard to determine whether the results are worse or better than the gold standard approach. The problem that remains is that it is not possible to perform measurements to separate model and measurement errors. Both errors occur simultaneously and cannot be eliminated beforehand so that only one error remains. Using synthetic data (e.g., generated using optimal control) is another way to validate novel approaches. A limitation of this approach lies within the aspect that measurement noise (especially STAs) cannot yet be simulated correctly. Up to now, validation remains a key challenge of musculoskeletal simulations.

Bailly et al. (2021) hypothesise, that tracking multimodal motion measurement can lead to smaller kinematic errors, thus handling the sim2real gap. The papers we reviewed which tracked multimodal motion measurement data supported this hypothesis. Each paper sorted into the *EMG-informed tracking* cluster uses a multimodal motion measurement and tracking approach. Kinematic errors were reported to be decreased using this approach in every publication but one. Mallat et al. (2021), categorised into the *Kalman Filter* cluster, fused RGB camera data and IMU sensor data to track human gait. They hypothesise that through this approach, individual weaknesses (marker occlusion and sensor drift) of the two measurement and sensor technologies compensate each other. Analogous, Pearl et al. (2023) fused camera and IMU data to track human gait. But instead of using a Kalman Filter, the authors used dynamic optimization to analyze experimentally measured motion. This multimodal approach outperformed single-modality approaches (using either only IMU or video data for human motion analysis). Halilaj et al. (2021) used both RGB camera data and IMU data in combination with a statistical shape model. Both the shape and the pose of the statistical shape model are optimized so that model joint centers best match estimated joint centers identified using the RGB camera. IMU and video data is then fused by adding an error term in the optimization minimizing the difference between video-based angular velocity and angular velocity values measured by the IMU sensors. Analogous to the results reported by Mallat et al. (2021), motion analysis performance was enhanced as the sensor technologies compensate each other’s weaknesses. Atrsaei et al. (2016) fused depth-camera data and IMU sensor data to track human arm motion. Again, the sensor fusion led to a decrease of the kinematic error. This is in line with a proposal we made in a prior publication that multimodal motion tracking could compensate IMU-sensor inherent problems like sensor drift or calibration problems in order to gain more reliable and accurate motion measurements (Wechsler et al., 2023).

It is important to note that the classification scheme presented in this review is not strictly selective, since the individual levels of the proposed clusters are not equal. For instance, some clusters describe the mathematical method that was used in simulations (*trajectory optimization* and *Kalman Filter*) whereas others directly describe the way how the sim2real gap is handled (*Minimization of kinematic error, BM parameter adjustment*, *computed quantities adjustment*). Many methods could therefore be fitted to various clusters. For example, every paper sorted into the *EMG-informed tracking* cluster uses *trajectory optimization*. The *EMG-informed tracking* cluster could therefore also be regarded as a subcategory of the *trajectory optimization* cluster. However, we decided to list these papers separately because tracking multimodal data (marker position/joint angle trajectories and EMG-measurements) is a distinguishing feature of these papers. The same applies for publications sorted into the *Kalman Filter* cluster since these could also be sorted into the *computed quantities adjustment cluster* or the *Minimization of kinematic error* cluster.

Additionally, there are limitations regarding the applied search strategy and the screening process. Both aspects are subjective to the decision of the authors. Even though inclusion and exclusion criteria were defined, objectivity cannot be guaranteed. Furthermore, the lack of consistent and uniform nomenclature complicates the identification of relevant papers using a search string. The term *sim2real gap* is not an established term in the field of biomechanical simulation but a description of the issue addressed in this review article. Therefore, searching for and identifying articles that handle the sim2real gap is laborious and error-prone. In addition, simulations that are free of residuals, either by definition (FD) or minimisation methods (ID), are sometimes called *dynamically consistent*. We decided against using this term, as there appears to be no clear definition and mutual understanding of the term in the biomechanical community. This circumstance has further complicated the search and selection of methods relevant for this review article. Consequently, even though the literature search was performed to the best of our knowledge, completeness of the review cannot be ensured.

## 5 Conclusion

This review identified and analyzed methods for handling the sim2real gap, the deviation between reality and musculoskeletal simulations which occurs because of kinematic and dynamic errors. The results showed that different solution approaches exist in literature, but there is no consensus on which method is most appropriate. Generally, FD approaches always generate simulations without residuals. However, to achieve this, the simulation may move far away from experimental measurements or IK results. This includes methods included in the *trajectory optimization*, *EMG-informed tracking* and *controller-based tracking* cluster. Therefore, the sim2real gap shows up as this deviation between the recorded movement and the simulated movement. An ID approach generates residual forces and torques while usually tracking experimental measurements more closely than FD approaches. Therefore, the sim2real gap shows up through the residuals. However, there are ways to reduce the residuals by adjusting either kinematic or dynamic simulation parameters. This includes methods included in the *BM parameter adjustment* and *computed quantities adjustment* cluster. Comparing the size of residuals to given recommendations provides information on the size of the sim2real gap. However, smaller residuals do not necessarily mean that the sim2real gap is smaller. In both methods, there is a risk of overfitting to the specific experimental data used, meaning that the adjusted parameters or quantities are unrealistic. Prevention methods should be taken to reduce this risk (e.g., constraining the solution space). By using a *Kalman Filter,* the kinematic error of simulation results is minimized as error models are used to compute optimal state estimations (e.g., marker positions, sensor positions or orientations). However, the quality of the optimized state vector estimation is restricted by the accuracy of the implemented error models. *Statistical approaches* minimize the kinematic error by maximizing a specific probability (e.g., maximum likelihood of a parameter’s posterior distribution) which leads to more accurate joint angle estimates in contrast to standard approaches for a two-dimensional movement. However, the methods are largely restricted by convergence problems and the required high computational effort. Ultimately, the method choice largely depends on various factors: available model, input parameters, investigated movement and of course the underlying research aim. However, we conclude that multimodal approaches tracking kinematic and dynamic measurements may be one possible solution to handle the sim2real gap as methods tracking multimodal measurements (some combination of sensor position/orientation or EMG measurements), consistently lead to better tracking performances. Initial analyses show that motion analysis performance can be enhanced by using multimodal measurements as different sensor technologies can compensate each other’s weaknesses (e.g., marker occlusion and IMU drift). FD approaches (*trajectory optimization*, *controller-based tracking*, *EMG-informed tracking*) or a Kalman Filter are suitable for the simultaneous processing of multimodal measurement data.

## Data Availability

The original contributions presented in the study are included in the article/Supplementary Material, further inquiries can be directed to the corresponding author.
