# Supplementary material for: Bridging the sim2real gap. Investigating deviations between experimental motion measurements and musculoskeletal simulation results—a systematic review
Source: Front Bioeng Biotechnol. 2024 Jun 11;12:1386874. doi: 10.3389/fbioe.2024.1386874 (PMC11196827; doi:10.3389/fbioe.2024.1386874)
Supplement: Supplementary file 1 [file DataSheet1.PDF]

Supplementary Material

1 Classification of identified publications

Table A 1: Classification of all identified publications to their respective solution cluster

Minimisation of kinematic error	BM parameter adjustment	Computed quantities adjustment	Trajectory optimization	Kalman Filter	EMG-informed tracking	Controller-based tracking	Statistical approach
Cockcroft Muller and Scheffer (2014)	Vaughan, Andrews and Hay (1982)	Koopman, Grootenboer and Jongh (1995)	Chao and Rim (1973)	Atrsaei, Salarieh and Alasty (2016)	Neptune and Hull (1998)	Thelen, Anderson and Delp (2003)	Lv, Chai and Xia (2016)
Allen et al. (2017)	Riemer and Hsiao-Wecksler (2009)	Kuo (1998)	Yamaguchi and Zajac (1990)	Ćesić et al. (2016)	Meyer et al. (2016)	Thelen and Anderson (2006)	Pataky, Vanrenterghem and Robinson (2019)
Begon et al. (2017)	Noamani et al. (2018)	Cahouët, Luc and David (2002)	Neptune (1999)	Bonnet et al. (2017)	Bélaise, Dal Maso, et al. (2018)	Seth and Pandy (2007)	
Laidig, Schauer and Seel (2017)	Faber, van Soest and Kistemaker (2018)	Mazzà and Cappozzo (2004)	Kaplan and Heegard (2001)	Joukov et al. (2017)	Bélaise, Michaud, et al. (2018)	Da Silva, Abe and Popović (2008)	
Schellenberg et al. (2017)	Fritz, Krökk and Schwameder (2019)	Muller, Pontonnier and Dumont (2018)	Koh and Jennings (2003)	Joukov et al. (2018)	Moissenet et al. (2019)	Ghafari, Meghdari and Vossoughi (2009)	
Niu et al. (2018)	Price et al. (2020)	Inai et al. (2020)	Menegaldo, Toledo Fleury and Weber (2006)	Yuan et al. (2019)	Bailly et al. (2021)	Remy and Thelen (2009)	
Tagliapietra et al. (2018)		Sturdy, Silverman and Pickle (2022)	van den Bogert, Blana and Heinrich (2011)	Joukov et al. (2020)	Ceglia, Bailly and Begon (2023)	Demircan et al. (2010)	
Bilesan et al. (2021)		Werling et al. (2023)	van den Bogert et al. (2012)	Sy, Lovell and Redmond (2020)		Watanabe and Sugi (2010)	
Halilaj et al. (2021)			Morrow et al. (2014)	(Mohammadi *et al.*, 2020)		Jackson, Hass and Fregly (2015)	
Al Borno et al. (2022)			Groote et al. (2016)	Mallat et al. (2021)		Mouzo et al. (2018)	
Zhou, Lannan and Fan (2022)			Lin and Pandy (2017)	Cuadrado et al. (2021)		Stanev and Moustakas (2018)	
Lefebvre et al. (2023)			Lin, Walter and Pandy (2018)	Sy, Lovell and Redmond (2021)		Maurice et al. (2019)	
			Falisse et al. (2019)	Lugrís et al. (2024)		Arash Haghpanah et al. (2022)	
			Dorschky et al. (2019)			Wang, Guo and Tian (2022)	

Minimisation of kinematic error	BM parameter adjustment	Computed quantities adjustment	Trajectory optimization	Kalman Filter	EMG-informed tracking	Controller-based tracking	Statistical approach
			Pallarès-Lopez et al. (2019)				
			Nitschke et al. (2020)				
			Dembia et al. (2020)				
			Febrer-Nafría et al. (2020)				
			Haralabidis et al. (2021)				
			Wang et al. (2021)				
			Febrer-Nafría, Fregly and Font Llagunes (2022)				
			Nitschke et al. (2023)				
			Pearl et al. (2023)				

2 Summarization of each method regarding the sim2real gap for every cluster

Table A2: Minimisation of kinematic error: method description, adjusted parameters and information regarding the accuracy of the method for every publication listed in the cluster

Author and Year	Method description	Kinematic variable	Evaluation/ Accuracy
Cockcroft et al. (2014)	Aim: IMU-based estimation of hip joint angles in cycling using a complementary filter<br>– Investigation of slow, medium and fast pedalling<br>– Complementary filter compensates dynamic acceleration in accelerometer signal of IMU to enhance the estimation of gravity<br>– Better estimation of gravity leads to more accurate orientation estimations	Sensor orientations	Validated using standard approach (MAE for three axes of hip joint angle):<br>– Slow: 2.1°<br>– Medium: 2.6°<br>– Fast 2.6°
Allen et al. (2017)	Aim: Accurate prediction of joint angles based on IMU-measurements using nonlinear state estimation technique<br>– Estimation of joint angles based on dynamical model of the knee joint<br>– Optimization of parameters of dynamic model needed<br>– Comparison of performance of proposed state dependent coefficient, extended Kalman Filter (EKF) and rotation matrix method (RMX)	Angular velocities, accelerations	Evaluated using reference angles from joint encoder. Mean RMSE (joint angle) for every estimation method:<br>– SDC: 2.70°<br>– RMX: 2.86°<br>– EKF: 4.42°
Begon et al. (2017)	Aim: Enhancement of humerus rotational kinematics using marker projections<br>– Projection of skin markers on longitudinal arm axes (desired axes) to compensate the effect of STAs<br>– Investigation of marker clusters (placed on cuffs) and single markers placed directly onto the skin<br>– Comparison of effect of marker selection for marker projection on quality of estimated humerus kinematics<br>– Best results achieved by either projecting marker clusters or selection of single markers	Marker positions	Validated using optical markers attached to cortical bone pins: Deviations reduced to mean value of 5°
Laidig et al. (2017)	Aim: Correction of heading error caused by magnetic interference in IMU-based motion analysis using kinematic constraints<br>– Method is specific to present kinematic constraint (joint type): only applicable for approximate hinge joints<br>– Correction of heading error by projecting joint axis vectors of two segments into plane of global coordinate system<br>– Without heading error, axes around which segments rotate should coincide<br>– With heading error, rotation axes are rotated relative to each other	Sensor orientations	Validated using simulated motion: Reduction of RMSE between reference and estimated joint angles from 26.0° (uncorrected) to 2.6° (corrected)

Author and Year	Method description	Kinematic variable	Evaluation/ Accuracy
	– Shift of projected axes describes heading error		
Schellenberg et al. (2017)	Aim: Investigating the effect of various scaling and kinematic weighting options on tracking performance<br>– Analysis of tracking performance of OpenSim's Inverse Kinematic method<br>– Two different scaling option:<br>– Standard marker-based OpenSim scaling approach<br>– Scaling approach based on the position of functional joint rotation axes and centers of rotation in addition to the standard approach<br>– Three different aspects for weighting options:<br>– In- or exclusion of position of functional axes and centers of rotation<br>– In- or exclusion of pre-calculated joint axes<br>– Different weightings on skin markers<br>– Generation of reference movement data based on the method of [List et al. 2013] determining the functional axes and centers of rotation followed by calculating joint angles and joint moments using a direct kinematic procedure and quasi-static inverse dynamics method, respectively	Marker positions	Evaluated using reference movement data. Best results are achieved by:<br>– Including functional axes and centers of rotation in scaling<br>– Including functional axes and centers of rotation in Inverse Kinematics method<br>– Including pre-calculated joint angles
Niu et al. (2018)	Aim: Estimation of lower-limb kinematics based on A-mode ultrasound data<br>– Combination of ultrasound transducer and optical markers attached on cuff<br>– Determination of ultrasound detected point (anatomical landmark) based on known position and pointing direction of ultrasound transducer relative to marker positions	Marker positions, ultrasound detected points	Validated using cortical bone pins as reference (RMSE) for rotations and translations respectively:<br>– Ultrasound: 3.44 °, 4,88 mm<br>– Skin markers: 6.32 °, 6.26 mm
Tagliapietra et al. (2018)	Aim: IMU-based estimation of kinematics<br>– Calibration of system using predefined static configuration with known joint angles: Alignment of each virtual sensor so that its orientation matches the orientation of the associated experimental sensor<br>– Orientation-based Inverse Kinematics method minimising difference between experimental and virtual sensor orientations	Sensor orientation	Validated using encoder and standard approach: RMSE (joint angles) < 6°
Bilesan et al. (2021)	Aim: Enhancement of Kinect-based joint angle estimation for lower limb tracking<br>– Tracking of virtual markers using the Kinect version 2<br>– Kinematic tracking with and without joint constraints<br>– Determination of joint angles by computing Euler angles rotating segment-based coordinate systems into each other	Marker positions	Validated using standard approach (mean RMSE for all joint angles)<br>– Without constraints: 2.49 °<br>– With constraints: 1.59 °
Halilaj et al. (2021)	Aim: Fusion of IMU and video data to enhance gait analysis<br>– Video-based estimation of 3D-joint centers<br>– Video-based joint angle estimation using statistical shape model: optimization of both shape and pose so that model joint centers best match estimated joint centers	Angular velocity	Mean error for angular velocity estimations between video and IMU-data:<br>– Without IMU-fusion: 1.53 ± 1.02

Author and Year	Method description	Kinematic variable	Evaluation/ Accuracy
	– Fusion of video and IMU-data by adding error term: Minimization of difference between video-based angular velocity and experimental IMU-angular velocity		– With IMU-fusion: 0.84 ± 0.44 rad/s
Al Borno et. al (2022)	Aim: IMU-based lower limb kinematics estimation in OpenSim<br>– Capable of drift compensation<br>– Inverse Kinematics approach: least squares method minimising difference between orientation of experimental and model fixed coordinate systems	Orientations	Validated using standard approach:<br>– Median RMSE between IMU and optical system between 3-6 °
Zhou et al. (2022)	Aim: Optimization of depth-camera based motion analysis<br>– Optimization of depth-camera based motions by creating smoother joint trajectories and including anthropometric constraints<br>– Denoising of depth-camera based kinematics using a Tobit particle filter for mitigating the effect of self-occlusion<br>– Maintaining constant bone lengths using a differential evolutionary algorithm to further enhance quality of tracking performance	Joint positions and angles	Validated using standard approach: Improvement of joint angle estimation by 40-60%
Lefebvre et al. (2023)	Aim: Assessing the effect of scapula marker weightings on estimated scapular kinematics<br>– Creation of reference model and reference data for optimization of scapula marker weightings<br>– Reference model: composed of thorax and scapular segments, all defined as free bodies with 6 degrees of freedom<br>– Reference data: created using scapular pin locator handled by expert examiner<br>– Optimization of marker weightings by minimization of difference between scapular orientation of reference model and multibody kinematics model	Marker weightings	Validated using reference model and data. Improvement of orientation estimation in the ranges of 0.9° - 12.1° using optimized marker weightings.

Table A 3: BM parameter adjustment cluster: method description, adjusted parameters and information regarding the accuracy of the method for every publication listed in the cluster

Author and Year	Method description	Adjusted Parameters	Evaluation/ Accuracy
Vaughan et al. (1982)	Aim: Selecting BSIPs based on kinematic data and optimization theory<br>– Minimization of difference between measured and calculated GRFs (expression of residuals)<br>– Adjustment of BSIPs using optimization<br>– Comparison of performance for three different movements (running, long jumping, kicking)	Mass, center of mass, centroid moment of inertia of 14 body segments (head, upper arms, forearms, hands, thighs, shanks, feet and trunk)	Improvement of accuracy of computed vertical GRFs for running (37 %), long jumping (33 %), kicking (26 %) against measurement data

Author and Year	Method description	Adjusted Parameters	Evaluation/ Accuracy
Riemer und Hsiao-Wecksler (2009)	Aim: Finding optimal set of subject-specific BSIPs to reduce errors in net joint torques<br>– Minimization of difference between measured and calculated GRFs (expression of residuals)<br>– Implementation of bi-level optimization<br>1. Simultaneous adjustment of BSIPs and segment-angle trajectories<br>2. Further optimization of BSIPs	Mass, center of mass and moment of inertia of shank, thigh, torso BSIPs	Validated on error-free reference data (constructed using anthropometric data and movement data); used to compute true GRFs<br>– Reduction of difference between true and calculated GRFs and torques to RMSE less than 0.02 N or 0.02 Nm<br>– Reduction of error in net joint torque due to BSP inaccuracies by 77 % (average RMSE over all joints)
Noamani et al. (2018)	Aim: Optimization-based, non-invasive method for estimation of subject-specific BSIPs in 3D kinetics of the HAT<br>– Minimization of difference between intersegmental trunk moments along spine computed by bottom-up and top-down approach (expression of residuals)<br>– Adjustment of trunk segment BSIPs	Joint center of rotation and center of mass of 6 thoracic segments	Reduction of net joint error (difference between intersegmental trunk moment along spine computed by bottom-up and top-down approach) by 79 % (median among 11 participants)
Faber et al. (2018)	Aim: Estimation of mechanically consistent kinematics while maintaining consistency to experimental kinematics<br>– Set up of constrained optimization problem<br>– Minimization of Euclidean distance between model and experimentally measured marker positions<br>– Residual forces and torques constrained to be zero	Measured marker trajectories	Complete removal of residual forces and torques by allowing small changes to measured marker positions (range of 1 cm)
Fritz et al. (2019)	Aim: Evaluating effect of BSIP estimation on ID results for specific user group (ski jumpers) for movement incorporating high accelerations (imitation jump)<br>– Minimization of difference between measured and calculated GRFs (expression of residuals)<br>– Adjustment of BSIPs of trunk and thigh segments using optimization	Mass, center of mass, moment of inertia of trunk and thigh segments	Improvement of accuracy of computed vertical GRF by 11 %
Price et al. (2020)	Aim: Optimization of model marker placement using dynamic trials to reduce human inconsistency from marker placement process<br>– Minimization of sum of squared difference between model and experimentally measured marker positions<br>– Adjustment of model marker placement for every time step of simulation	Model marker position	RMSE (marker tracking) decreased by 38 % (across all 8 participants and stride frequencies)

Table A4: Computed quantities adjustment cluster: method description, adjusted parameters and information regarding the accuracy of the method for every publication listed in the cluster

Author and Year	Method description	Adjusted parameters	Evaluation/ Accuracy
Koopman et al. (1995)	Aim: Estimation of non-measured rotations for reducing kinematic and dynamic errors of musculoskeletal simulations<br>– Minimization of imbalance moment in frontal plane<br>– Imbalance moment: ground reaction torques have to be zero, except for vertical component that describes friction between feet and floor (expression of residuals)<br>– Computation of non-measured rotation (hip abduction/ adduction and HAT rotation) to minimize imbalance moment	Non-measured rotations (hip abduction/ adduction and HAT rotation)	– Imbalance moment reduced<br>– Qualitative comparison of computed GRFs with data from literature: data shows characteristic patterns
Kuo (1998)	Aim: Precision improvement of ID computations<br>– Assembly of all available dynamic equations in overdetermined system<br>– Computation of joint torques by solving overdetermined system using pseudoinverse of matrix and optimization<br>– Computation of joint torques that best agree (in least squares sense) with both kinematic and kinetic measurements	– Ground reaction forces<br>– Angular accelerations	Validated using simulated data<br>– RMSE overall 34 % lower for joint torque estimation (compared to Newton-Euler method)<br>– Reduction of RMSE for joint accelerations by 30 %
Cahouët et al. (2002)	Aim: Accurate estimation of joint accelerations to enhance inverse dynamic results<br>– Set of equations relating body kinematics and force plate dynamics<br>– Set of equation relating angular accelerations to joint angle measurements<br>– Combination of the two set of equations leads to overdetermined system relating both measurement modalities (joint angles and force plate data) to joint accelerations<br>– Least squares estimation of joint accelerations that best agree with both measurements	Joint accelerations	Mean RMSE of 0.5 % between measured and calculated GRF
Mazzà und Cappozzo (2004)	Aim: Estimation of joint kinematics and kinetics using measured external resultant loads and readily available parameters<br>– Minimization of difference between measured and calculated GRFs (expression of residuals)<br>– Adjustment of measured segment-angle trajectories using optimization<br>– Constraint of beginning and end of movement with input data (segment-angle data) from measurements<br>– Variation of segment angle trajectories in between beginning and end of movement	Segment angle trajectories	RMS difference of 0.40 % over 10 trials between benchmark GRFs and estimated values
Riemer und Hsiao-Wecksler (2008)	Aim: Finding optimal angular position data for error reduction in joint torque estimation<br>– Minimization of least squares difference between measured and calculated GRFs (expression of residuals)<br>– Adjustment of segment-angle trajectories	Segment angle trajectories	Validated against reference values (synthetic data)<br>– Reduction of difference between true and computed GRFs to values less than 0.001 N or 0.001 Nm

Author and Year	Method description	Adjusted parameters	Evaluation/ Accuracy
			– Up to 79 % improvement for RMSE between true and computed values for joint torque
Samaan et al. (2016)	Aim: Optimization of tracking weights of residual reduction algorithm (RRA) implemented in OpenSim<br>– Comparison of three different optimization algorithms to compute optimal tracking weights<br>– Reduction of RMSE between experimental and simulated pelvic translations and rotations, lumbar and upper extremity rotation and residual forces and torques	Tracking weights	Reduction of residual forces and moments to 10 N and 18 Nm respectively
Muller et al. (2018)	Aim: Development of fast and quasi-optimal method for muscle force estimation<br>– Computation of muscle force sharing solution for each joint (activation ratio database)<br>– Two-step process: interpolation and correction step<br>– Interpolation of muscle forces based on activation ratio database for input motion<br>– Correction of interpolated muscle forces so that simulation adheres to overall dynamic equilibrium	Muscle forces	Validated on reference muscle forces determined using synthetic reference motion data and optimization approach<br>– Cross-correlation coefficients ranked between 0.89 to 0.99 for all muscles
Inai et al. (2020)	Aim: Computation of muscle excitations using numerical integration and optimization to compute muscle excitation patterns for accurate kinematics tracking<br>– Computation of desired (experimentally measured) generalized coordinates at future point in time<br>– Adjustment of joint acceleration values<br>– Replacement of proportional-differential (PD) controller of OpenSim CMC method	Joint accelerations	Validated using synthetic data<br>– Maximum kinematic tracking error: 0.04 – 0.07°
Sturdy et al. (2022)	Aim: Optimization of tracking weight values of RRA implemented in OpenSim<br>– Optimization of tracking weights of RRA through minimization of weighed sum of root-mean-squared residual forces and moments and sum of RMS kinematic tracking error<br>– Results in minimization of residual forces and moments while tracking experimental data	Tracking weights	Substantial reduction of all residuals except for F_z<br>– RMS forces < 1 % peak external force<br>– RMS moment < 0.35 % peak external force
Werling et al. (2023)	Aim: Automatic and standardized computation of human movement dynamics from motion data<br>– Model scaling and inverse kinematics: Minimization of deviations of estimated marker positions from experimentally measured marker positions while adjusting joint angles, body segment scaling parameters and model marker positions	– Joint angles<br>– Scaling parameters<br>– Model marker positions,<br>– System's center of mass,	Reduction of residual forces and torques compared to data evaluated by experts

Author and Year	Method description	Adjusted parameters	Evaluation/ Accuracy
	– Center of mass fitting: Adjustment of center of mass of the system to be consistent with experimentally measured GRFs<br>– Angular dynamics fitting: Adjustment of rotational generalized coordinates of model root segment to be consistent with experimentally measured GRFs<br>– Final optimization: Adjustment of segment masses, marker offsets, segment scale factors and joint coordinates to minimize residual forces at the pelvis without introducing large deviations from previously computed joint angle trajectories	– Rotational generalized coordinates of model root segment	

Table A5: Trajectory optimization cluster: method description, optimization variables and information regarding the accuracy of the method for every publication listed in the cluster

Author and Year	Method description	Cost function variables		Evaluation/ Accuracy
		Tracked	Minimized	
Chao und Rim (1973)	Aim: Determination of applied moments which produces observed gait motion<br>– Dynamic optimization of joint torques producing experimental joint angle trajectories<br>– Steepest descent optimization<br>– Adjusts both input and state variable<br>– Dynamically consistent over time	Joint angles	-	Set of applied moments for fictious subject seen as exact solution<br>– Computation of joint angle trajectories from known joint moments<br>– Computed moments are within 5 % of exact solution
Davy und Audu (1987)	Aim: Dynamic optimization for solving muscle redundancy problem<br>– Dynamic optimization for muscle force estimation for swing phase of gait<br>– Explicit multibody dynamics formulation<br>– Fletcher-Reeves conjugate-gradient algorithm<br>– FD approach: no undesired residuals<br>– Adjusts both input and state variable	Joint angles	Metabolic energy consumption	Qualitative evaluation of motion tracking performance
Yamaguchi und Zajac (1990)	Aim: Finding muscle excitation patterns needed for two-dimensional model to take a step<br>– Dynamic programming for muscle excitation estimation<br>– Two step control-estimation approach<br>– Coarse optimization (neglecting muscle activation and contraction dynamics)<br>– Fine-tuning (including muscle activation and contraction dynamics)<br>– No multibody dynamics formulation specified<br>– FD approach: no undesired residuals	– Joint angles<br>– Joint velocities	Cubed muscle stress	Qualitative evaluation of motion tracking performance

Author and Year	Method description	Cost function variables		Evaluation/ Accuracy
		Tracked	Minimized	
Neptune (1999)	Aim: Evaluation of optimization algorithm effect on performance of tracking problem<br>– Estimation of muscle controls producing pedalling motion<br>– Optimal control problem converted in parameter optimization problem (single shooting)<br>– Comparison of sequential quadratic programming method, downhill simplex method, simulated annealing<br>– FD approach: no undesired residuals<br>– Adjusts both input and state variable	– Horizontal and vertical pedal force<br>– Pedal angle<br>– Crank torque<br>– Joint moments	-	Reproduction of experimental data within 1 SD for all parameters
Kaplan und H. Heegaard (2001)	Aim: Fast optimal control solution algorithm for musculoskeletal simulation<br>– Estimation of neural excitations that produce steady state pedalling motion<br>– Direct collocation<br>– Dynamic equilibrium as constraint<br>– FD approach: no undesired residuals<br>– Adjusts both input and state variable	– Crank velocity<br>– Feet angles	Muscle activations	Qualitative evaluation of motion tracking performance and comparison between computed and measured muscle activations
Koh und Jennings (2003)	Aim: Dynamic optimization for joint torque estimation for complex, multiphase motions<br>– Dynamic optimization of joint torques producing experimental joint angle trajectories<br>– Optimal control problem converted in parameter optimization problem<br>– Adjusts both input and state variable	– Segment angles<br>– Center of mass location	-	Qualitative evaluation of simulated joint angles and computed joint torques
Menegaldo et al. (2006)	Aim: Dynamic estimation of muscle forces using optimal control<br>– Muscle redundancy problem formulated as optimal control problem (inverse dynamics optimal control)<br>– Sequential quadratic programming<br>– Dynamic estimation of muscle forces generates dynamic consistency over time<br>– Adjusts both input and state variable	Joint torques	-	– Forward dynamic optimal control as reference<br>– Torque fitting error (IDOC): 0.34 (sum of squared differences between true and computed joint torques)<br>– Torque fitting error (IDSO): 0.02
Van den Bogert et al. (2011)	Aim: Implicit model dynamics simulation for reduced computation time<br>– Motion data tracking using trajectory optimization<br>– Implicit multibody dynamics formulation<br>– Direct collocation<br>– Dynamic equilibrium as constraint<br>– FD approach: no undesired residuals<br>– Adjusts both input and state variable	– Joint angles<br>– Vertical and horizontal GRFs		Simulated joint angles and GRFs within half a standard deviation of normal gait

Author and Year	Method description	Cost function variables		Evaluation/ Accuracy
		Tracked	Minimized	
van den Bogert et al. (2012)	Aim: Development of optimal control approach for motion prediction<br>– Motion data tracking using trajectory optimization<br>– Implicit multibody dynamics formulation<br>– Direct collocation<br>– Dynamic equilibrium as constraint<br>– FD approach: no undesired residuals<br>– Adjusts both input and state variable	– Joint angles<br>– Vertical and horizontal GRFs	-	Qualitative evaluation of motion tracking performance
Morrow et al. (2014)	Aim: Comparison between static and dynamic muscle force estimation for upper extremity motions<br>– Dynamic optimization for muscle force estimation<br>– Simulated annealing<br>– Dynamic estimation of muscle forces generates dynamic consistency over time<br>– Adjusts both input and state variable	– Joint angles<br>– Handrim forces	-	Computed values (joint angles, handrim forces) within 1 SD of experimental data
Groote et al. (2016)	Aim: Comparison of effect of muscle dynamic optimization formulation on computation time and convergence for dynamic muscle force estimation<br>– Determination of muscle excitations producing specified joint torques (from ID)<br>– Most robust formulation: implicit muscle contraction dynamics<br>– Dynamical estimation of muscle forces generates dynamic consistency over time<br>– Ideal torque generators added to guarantee problem feasibility in the presence of modelling and measurement errors	-	Muscle excitations	Contribution of ideal torque generators < 0.7 Nm
Lin und Pandy (2017)	Aim: Full-body three-dimensional dynamic optimization simulation of human locomotion<br>– Dynamic optimization for muscle excitation estimation reproducing measured body motions and GRFs<br>– Explicit multibody dynamics formulation<br>– Direct collocation<br>– Dynamic equilibrium as constraint<br>– FD approach: no undesired residuals<br>– Adjusts both input and state variable	– GRFs<br>– Marker trajectories	Muscle activations	Validated against standard approach:<br>– RMSE: < 1.6 ° for rotations (across all subjects and degrees of freedom)<br>– RMSE: 0.6 cm for translations (across all subjects and degrees of freedom)
Lin et al. (2018)	Aim: Generation of predictive walking simulations at different speeds<br>– Dynamic optimization for muscle excitation estimation reproducing measured body motions and GRFs<br>– Explicit multibody dynamics formulation<br>– Direct collocation<br>– Dynamic equilibrium as constraint<br>– FD approach: no undesired residuals<br>– Adjusts both input and state variable	– GRFs<br>– Pelvis generalized coordinates	-	Validated against standard approach (RMSE):<br>– < 0.3° for pelvic rotations<br>– < 0.3 cm pelvic translation<br>– < 2.2° joint angles<br>– < 0.08 x bodyweight for GRFs

Author and Year	Method description	Cost function variables		Evaluation/ Accuracy
		Tracked	Minimized	
Falisse et al. (2019)	Aim: Demonstration of computational benefits of using algorithmic differentiation instead of finite-differences for optimal control approaches<br>– Dynamic optimization for muscle excitation estimation reproducing measured body motions and GRFs<br>– Implicit multibody dynamics formulation<br>– Direct collocation<br>– Dynamic equilibrium as constraint<br>– FD approach: no undesired residuals<br>– Adjusts both input and state variable	– Joint angles<br>– GRF<br>– GRT<br>– Joint torques of lower limbs, trunks and arm	Muscle activations	Accurate tracking of experimental walking data (average R^2: 0.95 +- 0.17)
Dorschky et al. (2019)	Aim: Estimation of kinematics and kinetics of raw inertial sensor data using dynamic optimization<br>– Dynamic optimization for muscle excitations reproducing measured body motions and GRFs<br>– Implicit multibody dynamics formulation<br>– Direct collocation<br>– Dynamic equilibrium as constraint<br>– FD approach: no undesired residuals<br>– Adjusts both input and state variable	IMU:<br>– Gyroscope<br>– accelerometer	Muscle excitations	Validated using standard approach:<br>– Gait kinematics: excellent correlation (≥ 0.93)<br>– Gait kinetics: strong correlation (≥ 0.90)
Pallarès-López et al. (2019)	Aim: Tracking experimental data without introducing high residuals using optimal control<br>– Dynamic optimization for joint acceleration and joint torque estimation reproducing measured body motions<br>– Limitation of residuals to tolerance level (± 2 N; ± 2 Nm)<br>– Implicit multibody dynamics formulation<br>– Multibody dynamics constraints: only relations between design variables (joint velocity is derivative of joint angle; joint acceleration is derivative of joint velocity)<br>– Adjusts both input and state variable	– Joint angles<br>– Joint velocities<br>– Joint accelerations<br>– Joint torques<br>– Three points along foot segment	-	Validated against standard approach: RMSE (joint angles) 1.97°
Nitschke et al. (2020)	Aim: Prediction of curved running using trajectory optimization and direct collocation method<br>– Dynamic optimization for muscle excitations reproducing measured body motions and GRFs<br>– Implicit multibody dynamics formulation<br>– Dynamic equilibrium as constraint<br>– FD approach: no undesired residuals<br>– Direct collocation<br>– Adjusts both input and state variable	– Marker trajectories<br>– GRFs	Muscle excitations	Validated using standard approach: joint angles and GRFs difference between simulated and experimental values generally less than 1 SD

Author and Year	Method description	Cost function variables		Evaluation/ Accuracy
		Tracked	Minimized	
Dembia et al. (2020)	Aim: Presentation and validation of OpenSim Moco: software toolkit for solving optimal control problems in OpenSim<br>– Explicit or implicit multibody dynamics formulation<br>– Dynamic equilibrium as constraint<br>– FD approach: no undesired residuals<br>– Direct collocation<br>– Adjusts both input and state variable	– Joint angles<br>– GRFs	– Muscle activation	Qualitative evaluation against experimental values
Febrer-Nafría et al. (2020)	Aim: Exploration of effect of direct collocation optimal control problem formulation on simulation convergence<br>– Dynamic optimization for joint accelerations or joint jerks reproducing measured body motions and GRFs<br>– Explicit multibody dynamics formulation<br>– Multibody dynamics constraints: only relations between design variables (joint velocity is derivative of joint angle; joint acceleration is derivative of joint velocity)<br>– Constraints set on residuals (half reduction and full reduction)<br>– Direct collocation<br>– Adjusts both input and state variable	Tracking two modalities with and without GRF-tracking:<br>– Marker trajectories<br>– Joint angles	– Joint accelerations<br>– Joint jerk	Report of RMSE of marker and joint coordinates, joint torques, GRFs and GRTs (between simulation and experimental values)<br>– Joint coordinates: 0.34 – 1.33 cm<br>– GRFs: 0.03 N – 8.06 N<br>– GRTs: 0.02 Nm – 0.46 Nm<br>– Angular joint coordinates: 0.77° – 2.31°<br>– Marker coordinates:<br>– 1.12 cm – 1.33 cm
Haralabidis et al. (2021)	Aim: Development and evaluation of optimal-control based framework for analysing sprinting<br>– Implicit multibody dynamics formulation<br>– Constraints set on pelvis residuals<br>– Dynamic optimization for muscle excitations reproducing measured body motions and GRFs<br>– Direct collocation<br>– Adjusts both input and state variable	– Joint angles<br>– GRFs<br>– Joint torques	– Muscle activations	Average RMSD between simulation and experimental data<br>– Pelvis angles: < 1°<br>– Pelvis translations: < 0.3 cm<br>– Joint angles: < 1°<br>– GRFs: 5.9 % - 11.4 % (BW*H)<br>– Net joint moments: 17.7 Nm<br>– Lower-limb joint moments: 23.6 Nm
Wang et al. (2021)	Aim: Estimation of spine kinematics and subject-specific mechanical properties of intervertebral joints using an optimal-control approach<br>– Dynamic optimization for joint segmental forces (produced by the muscles) reproducing experimental marker trajectories<br>– Dynamic equilibrium as constraint<br>– FD approach: no undesired residuals<br>– Direct collocation	Marker trajectories	Joint segmental forces	Average RMSD between simulation and experimental marker positions: 7.7 mm

**Author and Year**	**Method description**	**Cost function variables**		**Evaluation/ Accuracy**
		**Tracked**	**Minimized**	
	– Adjusts both input and state variable			
Febrer-Nafría et al. (2022)	Aim: Personalisation of pre-defined knee trajectory parameters of active knee-ankle-foot orthosis based on optimal control simulations<br>– Dynamic optimization for joint jerk and joint torque change reproducing measured body motions and joint torques<br>– Implicit multibody dynamics formulation<br>– Constraints set on residuals<br>– Direct collocation<br>– Adjusts both input and state variable	– Joint angles<br>– Joint torques	– Segment local angular momentum<br>– Joint mechanical power<br>– Knee motor torque<br>– Joint jerk<br>– Joint torque change	Report of RMSE of joint angles and GRFs (between simulation and experimental values)<br>– Joint angles: 5.34°<br>– GRFs: 55.40 N
Nitschke et al. (2023)	Aim: Feasibility investigation for 3D optimal control simulation reconstructing change of direction running motions by directly tracking marker and GRFs<br>– Dynamic optimization for muscle excitations reproducing measured body motions and GRFs<br>– Implicit multibody dynamics formulation<br>– Dynamic equilibrium as constraint<br>– FD approach: no undesired residuals<br>– Direct collocation<br>– Adjusts both input and state variable	Coordite tracking:<br>– Joint angles<br>– Global pelvis translation<br>– GRFs<br>– Marker tracking:<br>– Position of 42 markers<br>– GRFs	– Muscle excitations<br>– Torque controls (arms)	Validated using standard approach (RMSE):<br>Marker tracking:<br>– 9.5 mm marker position<br>– 3.5° joint angles<br>– Coordinate tracking:<br>– 16.8 mm marker positions<br>– 1.4° joint angles
Pearl et al. (2023)	Aim: Video and inertial sensing data fusion via dynamic optimization for investigating if multimodal approach outperforms single data methods<br>– Dynamic optimization for joint accelerations and joint torques reproducing measured body motions<br>– Explicit multibody dynamics formulation<br>– Direct collocation<br>– Dynamic equilibrium as constraint<br>– FD approach: no undesired residuals<br>– Adjusts both input and state variable	– Position of surface keypoints (extracted from video data)<br>– Inertial data (joint velocities and accelerations)	– Joint torques<br>– Residual forces	Validated against standard approach (RMSE), but reported comparison between single-modality methods and dynamically constrained fusion results:<br>Joint angles:<br>– Improved estimation of joint angles by 6.0° ± 1.2° over vision-only approach<br>– Joint center positions:<br>– Improved estimation of joint centers by 4.5 ± 2.8 cm over IMU-only approach

Table A6: Kalman Filter cluster: method description, Kalman Filter state vector variables and information regarding the accuracy of the method for every publication listed in the cluster

**Author and Year**	**Method description**	**Kalman filter state vector variables**	**Evaluation/ Accuracy**
Atrsaei et al. (2016)	Aim: Combination of IMU-based and depth-camera based motion measurements for the estimation of human arm kinematics<br>– IMUs: estimation of orientation of upper and lower arm<br>– Depth-camera: estimation of position of upper and lower arm<br>– Sensor data fusion leads to compensation of IMU-drift and errors occurring from joint occlusion<br>– Decreased RMSE of sensor fusion-based results in comparison to either IMU-based or depth-camera based motion analysis	Body segments' angular velocities and orientations	Validated using standard approach: RMSE between 1.72° - 24.64°
Ćesić et al. (2016)	Aim: Human motion analysis and estimation on Lie groups based on marker trajectories<br>– Application of extended Kalman Filter on Lie groups for motion estimation based on marker trajectories<br>– Comparison of tracking performance of conventional tracking system (CS), extended Kalman Filter (EKF) and Lie groups extended Kalman Filter (LG-EKF)<br>– Higher estimation accuracy of LG-EKF in comparison to CS and EKF	Joint angles, angular velocities and angular accelerations	Validation using standard approach: Average RMSE (marker position) for LG-EKF smaller than CS and EKF
Bonnet et al. (2017)	Aim: Real time estimation of kinematics and kinetics of human movement and minimization of STAs effect<br>– Fusion of kinematic and kinetic measurement data in Kalman Filter state vector<br>– Optimal tracking of STA affected marker-trajectories because of simultaneous modification of all state vector parameters (segment lengths and local position of markers not strictly constant)	– Joint angles, velocities and accelerations<br>– Segment geometric parameters<br>– Inertial parameters<br>– Marker positions	Validated using standard approach<br>– Average RMSE for GRFs: 3.4 N, 8.6 N, 9.5 N (F_x, F_y, M_z)<br>– Marker tracking: RMSEs between 0.01 and 0.98 rad for all joint angles
Joukov et al. (2017)	Aim: Human motion analysis and estimation on Lie groups based on IMU-measurements<br>– Application of extended Kalman Filter on Lie groups for motion estimation based on IMU-measurements<br>– Comparison of tracking performance of extended Kalman Filter (EKF) and Lie groups extended Kalman Filter (LG-EKF)<br>– Attachment of markers on IMU-sensors enables comparison of method results<br>– Higher estimation accuracy of LG-EKF in comparison to EKF	Joint angular velocities and angular accelerations	Validated using standard approach: Average RMSE (marker position) for LG-EKF (6.05 cm) smaller than for EKF (8.65 cm)
Joukov et al. (2018)	Aim: Lower body motion estimation during gait to extract performance criteria for physiotherapy<br>– Implementation of rhythmic extended Kalman Filter<br>– Combination of extended Kalman Filter and canonical dynamical system<br>– Addition of constant jerk assumption to improve Kalman Filter estimations<br>– For rhythmic motion: jerk is function of phase and frequency	Joint angles, angular velocities and angular accelerations	Validated using marker-based motion capturing and EKF<br>– Smaller or same level of RMSE joint angle values for REKF compared to EKF

Author and Year	Method description	Kalman filter state vector variables	Evaluation/ Accuracy
	– Function is learned by canonical dynamical system (learning of underlying harmonic Fourier series)		
Yuan et al. (2019)	Aim: Enhancement of IMU-based motion analysis for highly dynamic motions<br>– Analysis of three types of highly dynamic motions: jumping, running and leg swinging<br>– Incorporation of uncertainty model of active accelerations and magnetic disturbances to compensate inclination and heading errors<br>– Extended Kalman Filter used for estimating acceleration error and magnetic errors<br>– Correction of sensor orientation using estimated errors	Orientation errors, gyroscope bias and magnetic disturbances	Validated using standard approach. RMSE between reference and estimated joint angles ranges between: 2.0° - 4.39°
Joukov et al. (2020)	Aim: Full body motion estimation on Lie groups based on either marker or IMU measurements<br>– Extension of the work presented in Ćesić et al. (2016) and Joukov et al. (2017)<br>– Application of extended Kalman Filter on Lie groups for motion estimation<br>– Comparison of tracking performance of conventional tracking system (CS), extended Kalman Filter (EKF) and Lie groups extended Kalman Filter (LG-EKF)<br>– Higher estimation accuracy of LG-EKF in comparison to CS and EKF	Joint angles, velocities and accelerations	Validated using simulated marker data. Analysis of average mean absolute error (MAE)<br>– CS: 27.73 mm<br>– EKF: 14.83 mm<br>– LG-EKF: 14.74 mm
Sy et al. (2020)	Aim: Development of lie group extended Kalman Filter (EKF) for lower body kinematics estimation using three reduced sensor configuration (three IMUs)<br>– Computation of lower body kinematics using extended Kalman Filter<br>– Three IMUs strategically placed on the feet and pelvis<br>– Inter-IMU distance measurements as additional information<br>– Addition of model constraint update to generate physiologically plausible body segment motions	Position, orientation and velocity of body segments	Validated against standard ap-proach (knee and hip joint angles, respectively):<br>– RMSE: 7.6°±2.6, 6.6±2.7°<br>– Correlation coefficient: 0.95±0.03, 0.87±0.16
Mohammadi et al. (2020)	Aim: Simultaneous estimation of muscle forces and upper arm model states<br>– Simultaneous estimation of joint angles, joint velocities, muscle forces, joint torques and muscle states<br>– Generation of estimates that are consistent with systems model and measurements<br>– Maintenance of consistency if some values are inaccurate or go missing (e.g. because of marker occlusion)	Joint angles and velocities, muscle forces, joint torques and muscle states	Validated against simulated data (generated using feedback linearization controller)<br>– Standard deviations of estimation error smaller than 0.07° for joint angles
Mallat et al. (2021)	Aim: Estimation of lower-limb joint kinematics using IMU and RGB camera data<br>– Combination of RGB-camera data and IMU data<br>– Using three visual inertial measurement units (combination of visual marker and IMU)<br>– Fusion of available data from both measurements for optimal lower limb estimation<br>– Hypothesis: measurement modalities counteract each other's weaknesses (sensor drift and marker occlusion)	Joint angles, velocities and accelerations	Validated against standard ap-proach: RMSE (joint angles): 3.5°
Cuadrado et al. (2021)	Aim: Estimation of full-body kinematics using an extended Kalman Filter	Position and velocity of each degree of freedom:	Evaluation of computed accelerations: RMSE between

Author and Year	Method description	Kalman filter state vector variables	Evaluation/ Accuracy
	– Tuning filter parameters for motion data captured using optical systems based on accelerations measured by IMU-sensors<br>– Kalman filter estimates body segment orientations and translations in case of pelvis body segment<br>– IMU-based accelerations are used to tune filter parameters so that difference between accelerations based on optical motion capture data and IMU-based accelerations is minimized	– Pelvis translations<br>– Relative angles at toes and neck<br>– Absolute orientation of remaining rigid bodies	IMU-based and estimated accelerations between 0.6 $m/_{s^2}$ and 1.2 $m/_{s^2}$
Sy et al. (2021)	Aim: Further development of lie group constrained extended Kalman Filter (EKF) for lower body kinematics estimation using reduced sensor configuration (three IMUs)<br>– Extension of Sy et al. (2020) by additional tracking of feet segments<br>– Computation of lower body kinematics using extended Kalman Filter<br>– Inter-IMU distance measurements as additional information<br>– Addition of model constraint update to generate physiologically plausible body segment motions<br>– Comparison of tracking performance based on two or three IMUs	Position, orientation and velocity of body segments	Validated against standard approach (Mean RMSE for joint angles)<br>– Three IMUs: 12.71° ± 1.60°<br>– Two IMUs: 13.43° ± 1.89°
Lugrís et al. (2023)	Aim: Real-time musculoskeletal analysis of experimental motion data<br>– Computation of full-body kinematics using extended Kalman Filter<br>– Extension of presented Kalman Filter in Cuadrado et al. (2021) by addition of accelerations in state vector	Position, velocity and acceleration for every degree of freedom	No quantitative or qualitative analysis of kinematic data.

Table A7: EMG-informed tracking cluster: method description, optimization variables and information regarding the accuracy of the method for every publication listed in the cluster

Author and Year	Method description	Cost function variables		Evaluation/ Accuracy
		Tracked	Minimized	
Neptune und Hull (1998)	Aim: Development of a forward dynamical model of cycling and optimization framework to simulate pedalling motion<br>– Comparison of different cost functions<br>– Best results achieved for tracking all available kinematic and kinetic parameters<br>– Tracking of muscle activity onset and offset enhanced consistency between EMG-measurement values from literature and calculated muscle activity but increased RMSE for other tracked variables<br>– Optimal control problem converted in parameter optimization problem (single shooting)<br>– Simulated annealing algorithm<br>– FD approach: no undesired residuals<br>– Adjusts both input and state variables	– Vertical and horizontal pedal force,<br>– Pedal angle,<br>– Crank torque<br>– Hip, knee, ankle torque	-	Best results: computed values within 1 SD of experimental values
Meyer et al. (2016)	Aim: Evaluation of patient-specific synergy-controlled neuromuscular simulation framework to predict walking motions for individuals after stroke<br>– Motion data tracking using trajectory optimization<br>– Addition of algebraic path constraints to constrain residual pelvis load (convergence tolerance: 1 N, 0.1 Nm) in each time step<br>– Implicit multibody dynamics optimization<br>– Direct collocation	– Upper body joint angles<br>– Lower body joint torques<br>– Muscle activations<br>– GRFs	Joint jerk	Qualitative evaluation of motion tracking performance
Bélaise et al. (2018a)	Aim: Forward dynamics-based optimisation for estimating the upper-limb muscle forces tracking both EMG and marker-data<br>– Implementation of muscle co-contraction<br>– Comparison between EMG and marker tracking, marker tracking and static optimization<br>– Explicit multibody dynamics formulation<br>– Direct multiple shooting<br>– FD approach: no undesired residuals<br>– Adjusts both input and state variables	– Marker trajectories<br>– EMG measurements	Muscle excitations	Validated using simulated data (using direct multiple shooting)<br>– EMG-marker tracking error: 0.31 (marker tracking: 0.43; static optimization: 0.17)

Author and Year	Method description	Cost function variables		Evaluation/ Accuracy
		Tracked	Minimized	
Bélaise et al. (2018b)	Aim: Determination of best tracking objective-function to accurately predict upper-limb muscle forces<br>– Comparison of effect of different cost functions on simulation results<br>– Lowest RMSEs achieved by cost functions tracking joint angles directly both with and without additional EMG-tracking<br>– Direct multiple shooting<br>– FD approach: no undesired residuals<br>– Adjusts both input and state variables	Tracking of different variables with and without EMG-tracking<br>– Joint angles<br>– Joint torques<br>– Marker trajectories		Validated using simulated data<br>– Lowest RMSE for directly tracking joint angles (1.57 ° without marker tracking and 1.67 ° with marker tracking)
Moissenet et al. (2019)	Aim: Adaptation of EMG-marker tracking optimization process to lower limb model during equinus gait<br>– Enforcement of physiologically generalized forces working on the pelvis to compensate missing upper part of the body and contralateral limb<br>– Explicit multibody dynamics formulation<br>– Direct multiple shooting<br>– FD approach: no undesired residuals<br>– Adjusts both input and state variables	– Marker trajectories<br>– Muscle excitations<br>– GRFs	Muscle activations	Qualitative evaluation on experimental data
Bailly et al. (2021)	Aim: Real time muscle force estimation using optimal control approach<br>– Comparison of tracking only marker trajectories with simultaneous tracking of marker trajectories and muscle excitations<br>– Explicit multibody dynamic formulation<br>– Moving horizon estimation algorithm<br>– FD approach: no undesired residuals<br>– Adjusts both input and state variables	Joint kinematics<br>(Muscle activations)	Muscle activations<br>(Muscle excitations)	RMSEs on joint kinematics almost always smaller when tracking both modalities
Ceglia et al. (2023)	Aim: Real-time muscle force estimation using optimal control approach<br>– Application of previously presented moving horizon estimation algorithm on experimental motion data; builds on work of Bailly et al. (2021)<br>– Explicit multibody dynamic formulation<br>– Moving horizon estimation algorithm<br>– FD approach: no undesired residuals<br>– Adjusts both input and state variables	– Marker trajectories<br>– Muscle activations	– Additional joint torques<br>– Muscle activations<br>– Joint angles<br>– Joint velocities	RMSE between experimental and estimated marker positions of 24.8 ± 6.7 mm and 20.3 ± 7.5 mm for two participants respectively

Table A8: Controller-based tracking cluster: method description, applied controller and information regarding the accuracy of the method for every publication listed in the cluster

Author and Year	Method description	Controller	Validation/ Evaluation/ Accuracy
Thelen et al. (2003)	Aim: Determination of muscle excitations driving muscle-actuated model to track experimental data<br>– CMC (computed muscle control)<br>– Computation of muscle excitations that drives musculoskeletal model towards desired (experimentally measured) kinematics using linear feedback controller<br>– FD approach: no undesired residuals	Linear feedback controller	Simulated pedal angles, crank angles and pedal forces within one SD of experimentally measured values
Thelen und Anderson (2006)	Aim: Generation of human walking simulations closely tracking body kinematics and GRF measurements<br>– Revision of CMC method (still different to CMC that is now implemented in OpenSim)<br>– Replacement of linear feedback controller with PD controller<br>– Implementation of residual elimination algorithm (REA)<br>– REA: computation of novel pelvic translations and lower back angular trajectories to reduce residuals<br>– FD approach	PD-controller	Small deviations from experimental kinematics and GRFs<br>– Mean RMSE for joint angles: 0.1 – 1.0° for half gait cycle<br>– Mean RMSE for GRF: < 7 N
Seth und Pandy (2007)	Aim: Estimation of joint torques and muscle forces based on motion tracking and neuromuscular tracking for accurate forward simulations of human movement<br>– Computation of joint torques based on observed kinematics and GRFs, tracking both parameters concurrently<br>– Computation of muscle excitations and muscle forces resulting in previously estimated joint torques<br>– FD approach	Feedback linearization	No quantitative evaluation/ validation
Da Silva et al. (2008)	Aim: Estimation of control signals tracking experimentally measured motion data<br>– Computation of control signal (joint torques) tracking desired (experimentally measured) system states defined by joint angles and joint velocities<br>– Control signal consisting of two components<br>– Predictive component (MPC): computes control signal tracking reference data<br>– Feedback component (PD-controller): compensates drift from MPC due to dynamic constraints<br>– Computation of joint torques and external forces that best achieve desired states<br>– FD approach	– Model predictive control (MPC)<br>– PD-controller	Evaluated using standard approach: Squared tracking error values between experimental and simulated state vectors between 0-0.4
Blana et al. (2009)	Aim: Estimation of muscle activities for functional electrical simulation to track experimental kinematics<br>– Two component controller: feedforward part (ANN) and feedback part (PID-controller in series with ANN)<br>– Computation of muscle activations tracking desired kinematics	– Artificial Neural Network (ANN)<br>– PID-controller	Evaluated using standard approach. RMSE between experimental and simulated shoulder and wrist joint angles respectively:<br>– Feedforward: 8.3°, 8.4<br>– Feedback: 4.6°, 4.0

Author and Year	Method description	Controller	Validation/ Evaluation/ Accuracy
	– Feedforward part: imitates inverse-dynamics model; generation of muscle activations required for desired motion<br>– Feedback part: outputs of PID-controller as inputs for ANN; generation of muscle activations<br>– Comparison of performance of feedforward controller, feedback controller and combination of both parts<br>– FD approach: no undesired residuals		– Combination: 3.3°, 3.7
Ghafari et al. (2009)	Aim: Identifying optimal control parameters for forward dynamic simulations using dynamic optimization<br>– Optimization of PID-controller parameters for optimal tracking of experimental kinematics and kinetics of hip, knee and ankle joints, pelvis and HAT (joint angles, joint torques, GRFs, pelvis and HAT position and orientation<br>– Optimization of control parameters by application of iterative feedback tuning method<br>– Computation of joint torques resulting in experimental kinematics<br>– FD approach: no undesired residuals	PID-controller	No quantitative tracking evaluation. Qualitative comparison between experimental and simulated results showed good correspondence
Remy und Thelen (2009)	Aim: Generation of kinematics and kinetics satisfying whole body equations of motion that best agree with measured motion and external data<br>– Novel residual elimination algorithm (REA)<br>– Computation of desired accelerations: generation of simulated motion consistent with measured GRFs with motion staying as close as possible to measured kinematics<br>– Adjustment of joint kinematics and GRFs using optimization; constraints to reduce residuals<br>– FD approach	PD-controller	Validated using synthetic and experi-mental data<br>– Synthetic data with noise: elimination of residuals; enhancement of joint moment computation by 23 % (hip) and 59 % lower back<br>– Synthetic data with noise and offset: no quantitative values<br>– Experimental data: elimination of residuals; mean kinematic changes < 1°
Demircan et al. (2010)	Aim: Tracking of dynamic motions using a direct marker tracking approach<br>– Tracking of desired marker positions, velocities and accelerations<br>– Task space reconstruction methods<br>– Assignment of all markers to various tasks with different priorities<br>– Tracking of root segment and end effectors using higher priority marker tasks<br>– Lower priority marker tasks ensuring motion of remaining segments consistent with marker trajectory constraints<br>– FD approach: no undesired residuals	PID-controller	Evaluated using standard approach: difference between measured and simulated marker position between 0-4 cm
Watanabe et al. (2010)	Aim: Development of control strategy for functional electrical simulation to track experimental kinematics<br>– Computation of stimulation signals (muscle activations) to track wrist joint movement using PID-controller, inverse statics model (ISM) and inverse dynamics model (IDM)<br>– Incorporation of muscle activation dynamics<br>– FD approach: no undesired residuals	– Feedback controller: PID-controller<br>– Feedforward controller: ISM, IDM	Mean deviation between desired and tracked joint angles smaller than 3°

Author and Year	Method description	Controller	Validation/ Evaluation/ Accuracy
Jackson et al. (2015)	Aim: Enhancement of REA algorithm to achieve gait motions consistent with measured GRFs and accurate foot tracking motion<br>– Advancement of REA to track foot kinematics more closely<br>– Implementation of four aspects: manual modification of tracked marker weights, automatic modification of algorithm feedback gains, automatic calibration of model joint and inertial parameter values<br>– FD approach	PD-controller	– Elimination of residuals<br>– Lower marker tracking errors compared to original REA<br>– For feet: RMSE 3.1 ° vs 18.4 °
Mouzo et al. (2018)	Aim: Exploration of available control-based options to carry out FD simulation approaches for healthy and assisted gait analysis<br>– FD simulation approach for underactuated system<br>– Number of controllers is smaller than number of degrees of freedom (joint angles and cartesian coordinates of pelvis segment)<br>– Torque actuators for each joint<br>– Computation of net joint torques<br>– Tracking of experimental joint angles and cartesian coordinates of pelvis segment<br>– FD approach: no undesired residuals	– PD-controller<br>– Computed torque control	RMSE between FD and IDA:<br>– 0.0476 m for translational coordinates<br>– 0.0117 rad for angular coordinates<br>– 110.84 N for force components of resultant external forces<br>– 14.08 Nm for moment compo-nents of external reaction<br>– 14.66 for internal net joint torques
Stanev und Moustakas (2018)	Aim: Estimation of joint torques tracking experimental data using task goals<br>– Construction of task goals based on optical marker data<br>– Computation of generalized forces (joint torques) actuating the model<br>– Task goal tracking using generalized forces<br>– Estimation of muscle activations resulting in generalized forces using optimization<br>– FD approach: no undesired residuals	PD-controller	Kinematic RMSE < 2 cm
Maurice et al. (2019)	Aim: Assessment and enhancement of human motion performance<br>– LQR-controller computes motion of digital human model taking into account various tasks<br>– Motion tracking implemented in an operational acceleration task; tracking of desired (experimentally measured) accelerations<br>– Desired accelerations computed using PD-controller	– LQR-controller<br>– PD-controller	RMSE between simulated and experimental data: Smaller than 3 cm for all but two markers
Arash Haghpanah et al. (2022)	Aim: Development of control strategy for functional electrical simulation to track experimental kinematics<br>– Computation of muscle activation to directly track ankle joint movement using PD-controller<br>– Incorporation of muscle activation and deactivation dynamics<br>– FD approach: no undesired residuals	– PD-controller<br>– Sliding mode controller	No quantitative evaluation/ validation
Wang et al. (2022)	Aim: Computation of muscle forces and joint torques taking muscle contraction dynamics and force equilibrium within the musculotendon unit into account<br>– Forward-muscular-inverse-skeletal (FMIS) algorithm	Linear proportional feedback controller	Qualitative comparison of joint torques computed by SO and FMIS algorithm

Author and Year	Method description	Controller	Validation/ Evaluation/ Accuracy
	– Computation of joint torques using ID<br>– Computation of initial muscle activations using SO<br>– FD computation of joint torques based on SO results<br>– Addition of torque tracking term to static optimization cost function to compute FMIS joint torques<br>– Incorporation of muscle dynamics		

Table A9: Statistical approach cluster: method description, applied statistical approach and information regarding the accuracy of the method for every publication listed in the cluster

Author and Year	Method description	Statistical approach	Evaluation/ Accuracy
Lv et al. (2016)	Aim: Data-driven estimation of contact forces and internal torques from kinematic input data<br>– Building database including kinematic and kinetic motion data for one subject (prior term for newly measured motions)<br>– Minimization of objective function including different terms: friction cone term, prior term, physical term, data term and smoothness term.<br>– Physical term measures how well generated contact information and internal joint torques satisfied dynamic equilibrium	Maximum a posteriori framework	Evaluated against standard approach: Qualitative comparison of joint torque trajectories for both approaches
Pataky et al. (2019)	Aim: Enhancement of IK results through Bayesian approach<br>– Minimization of kinematic error for joint angle estimation using Bayesian IK<br>– Bayesian IK: specifically maximizes probability that kinematic parameters (joint angles and also joint rotation centers) yield measured (observed) marker trajectories	Bayesian statistics	More accurate IK estimates in more than 95 % of simulation results (compared to standard approach)

3 Experimental measurements collected in every publication

Table A10: Experimental measurements collected in every publication

Publication	Kinematic measurements	Kinetic measurements	Other measurements
Lugrís et al. (2024)	Marker trajectories	GRFs	-
Ceglia, Bailly and Begon (2023)	Marker trajectories	-	EMG
Lefebvre et al. (2023)	Marker trajectories<br>Scapula locator pin positions	-	-
Nitschke et al. (2023)	Marker trajectories	GRFs	-
Pearl et al. (2023)	RGB video data<br>IMU data<br>(Publicly available dataset from Trumble et al. (2017))	-	-
Werling et al. (2023)	Marker trajectories<br>(taken from Uhlrich et al. (2023) and Hamner and Delp (2013))	GRFs<br>(taken from Uhlrich et al. (2023) and Hamner and Delp (2013))	-
Al Borno et al. (2022)	Marker trajectories<br>IMU data	-	-
Arash Haghpanah et al. (2022)	Marker trajectories	GRFs	-
Febrer-Nafría, Fregly and Font Llagunes (2022)	Marker trajectories	GRFs<br>Crutch forces	
Sturdy, Silverman and Pickle (2022)	Marker trajectories<br>(Publicly available dataset from OpenSim)	GRFs<br>(Publicy available dataset from OpenSim)	-
Wang, Guo and Tian (2022)	Marker trajectories<br>Video data	-	EMG
Zhou, Lannan and Fan (2022)	Marker trajectories<br>Depth camera data<br>(Additional data taken from Ofli et al. (2013))	-	-
Bailly et al. (2021)	-	-	-
Bilesan et al. (2021)	Marker trajectories<br>Depth camera data	-	-
Halilaj et al. (2021)	IMU data<br>RGB video data	-	-
Haralabidis et al. (2021)	Marker trajectories	GRFs	EMG
Mallat et al. (2021)	IMU data<br>Marker trajectories<br>Augmented reality marker	-	-

Sy, Lovell and Redmond (2021)	Marker trajectories<br>IMU data<br>(taken from Trumble et al. (2017) and Sy et al. (2021))		
Wang et al. (2021)	Marker trajectories		X-rays
Dembia et al. (2020)	Marker trajectories	GRFs	EMG
Febrer-Nafría et al. (2020)	Marker trajectories	GRFs	-
Inai et al. (2020)	Marker trajectories	-	-
Joukov et al. (2020)	Marker trajectories		
Mohammadi et al. (2020)	-	-	-
Nitschke et al. (2020)	Marker trajectories	GRFs	-
Price et al. (2020)	Marker trajectories	GRFs	
Sy, Lovell and Redmond (2020)	Marker trajectories<br>IMU data	-	-
Dorschky et al. (2019)	IMU data<br>Marker trajectories	GRFs	-
Falisse et al. (2019)	Marker trajectories	GRFs	-
Fritz, Kröll and Schwameder (2019)	Marker trajectories	GRFs	-
Marurice et al. (2019)	Marker trajectories	GRFs<br>Drill forces	-
Moissenet et al. (2019)	Marker trajectories	GRFs	EMG
Pallarès-López et al. (2019)	Marker trajectories	-	-
Pataky, Vanrenterghem and Robinson (2019)	-	-	-
Yuan et al. (2019)	Marker trajectories<br>IMU data	-	-
Bélaise, Michaud, et al. (2018)	Marker trajectories	-	-
Bélaise, Dal Maso, et al. (2018)	-	-	-
Faber, van Soest and Kistemaker (2018)	Marker trajectories	GRFs	-
Joukov et al. (2018)	IMU data<br>Marker trajectories	-	
Lin, Walter and Pandy (2018)	Marker trajectories	GRFs	-
Mouzo et al. (2018)	Marker trajectories	GRFs	
Muller, Pontonnier and Dumont (2018)	-	-	-
Niu et al. (2018)	Marker trajectories		Ultrasound

Noamani et al. (2018)	Marker trajectories	GRFs	-
Stanev and Moustakas (2018)	-	-	-
Tagliapietra et al. (2018)	Marker trajectories<br>IMU data	-	-
Allen et al. (2017)	IMU data<br>Knee angle (from encoder)	-	-
Begon et al. (2017)	Marker trajectories	-	-
Bonnet et al. (2017)	Marker trajectories	-	-
Laidig, Schauer and Seel (2017)	-	-	-
Lin and Pandy (2017)	Marker trajectories	GRFs	EMG<br>Knee contact forces
Schellenberg et al. (2017)	Marker trajectories	GRFs	-
Atrsaei, Salarieh and Alasty (2016)	IMU data<br>Depth camera data	-	-
Groote et al. (2016)	Marker trajectories (taken from Model folder installed with OpenSim 3.2)	-	-
Lv, Chai and Xia (2016)	Marker trajectories	GRFs	-
Meyer et al. (2016)	Marker trajectories	GRFs	EMG
Samaan et al. (2016)	Marker trajectories	GRFs	-
Jackson, Hass and Fregly (2015)	Marker trajectories	GRFs	-
Cockcroft, Muller and Scheffer (2014)	Marker trajectories<br>IMU data	-	-
Morrow et al. (2014)	Marker trajectories<br>Wheel angle (wheelchair)	Handrim kinetics (wheelchair)	-
van den Bogert et al. (2012)	Marker trajectories	GRFs	-
Van den Boger and Heinrich (2011)	Marker trajectories<br>(taken from Winter (1991))	GRFs	-
Watanabe and Sugi (2010)	-	-	-
Blana, Kirsch and Chadwick (2009)	-	-	-
Ghafari, Meghdari and Vossoughi (2009)	Marker trajectories<br>(taken from Riener, Rabuffetti and Frigo (2002))	GRFs<br>(taken from Riener, Rabuffetti and Frigo (2002))	-

Remy and Thelen (2009)	Marker trajectories	GRFs	-
Riemer and Hsiao-Wecksler (2009)	Marker trajectories	GRFs	-
Da Silva, Abe and Popović (2008)	Marker trajectories (data taken from website that is not reachable anymore)	-	-
Riemer, Hsiao-Wecksler and Zhang (2008)	Marker trajectories	GRFs	-
Seth and Pandy (2007)	-	-	-
Menegaldo, Toledo Fleury and Weber (2006)	-	-	-
Thelen and Anderson (2006)	Marker trajectories	GRFs	
Mazzà and Cappozzo (2004)	Marker trajectories	GRFs	-
Thelen, Anderson and Delp (2003)	Crank angles	Pedal forces	
Koh and Jennings (2003)	Joint angle trajectories	-	-
Cahouët, Luc and David (2002)	Marker trajectories	GRFs	
Kaplan and Heegard (2001)	Pedal angles (taken from (Ting *et al.*, 1999))	-	-
Neptune (1999)	Crank and pedal angular displacement<br>Video data	Pedal force	
Kuo (1998)	Marker trajectories	GRFs	-
Neptune and Hull (1998)	Marker trajectories	Pedal force	
Koopman, Grootenboer and Jongh (1995)	Joint angle trajectories	-	-
Yamaguchi and Zajac (1990)	-	-	-
Davy and Audu (1987)	Joint angle trajectories (taken from Mann, Hagy and Simon (1975))	-	-
Vaughan, Andrews and Hay (1982)	Video data	GRFs	
Chao and Rim (1973)	Joint angle trajectories (taken from Eberhart (1947))	GRFs	
